# Hot carbonates deep within the Chicxulub impact structure

**DOI:** 10.1093/pnasnexus/pgad414

**Published:** 2024-01-11

**Authors:** Pim Kaskes, Marta Marchegiano, Marion Peral, Steven Goderis, Philippe Claeys

**Affiliations:** Research Unit: Archaeology, Environmental Changes and Geo-Chemistry (AMGC), Vrije Universiteit Brussel, 1050 Brussels, Belgium; Laboratoire G-Time, Université Libre de Bruxelles, 1050 Brussels, Belgium; Research Unit: Archaeology, Environmental Changes and Geo-Chemistry (AMGC), Vrije Universiteit Brussel, 1050 Brussels, Belgium; Department of Stratigraphy and Paleontology, University of Granada, 18071 Granada, Spain; Research Unit: Archaeology, Environmental Changes and Geo-Chemistry (AMGC), Vrije Universiteit Brussel, 1050 Brussels, Belgium; CNRS, Bordeaux INP, EPOC, UMR 5805, Université de Bordeaux, F-33600 Pessac, France; Research Unit: Archaeology, Environmental Changes and Geo-Chemistry (AMGC), Vrije Universiteit Brussel, 1050 Brussels, Belgium; Research Unit: Archaeology, Environmental Changes and Geo-Chemistry (AMGC), Vrije Universiteit Brussel, 1050 Brussels, Belgium

**Keywords:** clumped isotopes, Chicxulub, decarbonation, back-reaction, impactites

## Abstract

Constraining the thermodynamic conditions within an impact structure during and after hypervelocity impacts is extremely challenging due to the transient thermal regimes. This work uses carbonate clumped-isotope thermometry to reconstruct absolute temperatures of impact lithologies within and close to the ∼66 Myr old Chicxulub crater (Yucatán, México). We present stable oxygen (δ^18^O), carbon (δ^13^C), and clumped-isotope (Δ_47_) data for carbonate-bearing impact breccias, impact melt rock, and target lithologies from four drill cores on a transect through the Chicxulub structure from the northern peak ring to the southern proximal ejecta blanket. Clumped isotope-derived temperatures (*T*(Δ_47_)) are consistently higher than maximum Late Cretaceous sea surface temperatures (35.5°C), except in the case of Paleogene limestones and melt-poor impact breccias outside of the crater, confirming the influence of burial diagenesis and a widespread and long-lived hydrothermal system. The melt-poor breccia unit outside the crater is overlain by melt-rich impact breccia yielding a much higher *T*(Δ_47_) of 111 ± 10°C (1 standard error [SE]), which likely traces the thermal processing of carbonate material during ejection. Finally, *T*(Δ_47_) up to 327 ± 33°C (1 SE) is determined for the lower suevite and impact melt rock intervals within the crater. The highest temperatures are related to distinct petrological features associated with decarbonation and rapid back-reaction, in which highly reactive CaO recombines with impact-released CO_2_ to form secondary CaCO_3_ phases. These observations have important climatic implications for the Cretaceous–Paleogene mass extinction event, as current numerical models likely overestimate the release of CO_2_ from the Chicxulub impact event.

**Significance Statement**

For the first time in the field of impact geology, clumped-isotope analyses are used to confirm the presence of carbonate phases formed at high temperature (>100°C) deep within a large terrestrial impact structure. Despite extensive hydrothermal overprinting, hot signatures of early thermal impact processes are still preserved in suevite and impact melt rock from within and outside the Chicxulub impact structure in México. The impactites recording the highest temperatures show petrological characteristics akin to thermal decarbonation, derived from shock-devolatilization of Yucatán carbonate target rocks, followed by back-reaction of CaO with impact-released CO_2_. Our isotopic and petrological data ground-truth current-state thermodynamic models of impact-induced hydrothermal systems and proximal ejecta processes, and it places crucial new constraints on the release of volatiles across the Cretaceous–Paleogene boundary.

## Introduction

The ∼200 km wide Chicxulub impact structure in México, formed by a hypervelocity impact event ∼66 Myr ago, constitutes a natural laboratory to examine the response of distinct lithological units to extreme shock conditions (1–6). Constraining the rapid temperature changes that target rocks experience during and after impact cratering events remains extremely challenging due to generally poorly preserved impact lithologies and the complexities of reconstructing high-temperature conditions in a laboratory setting (7, 8). Over the last 15 years, carbonate clumped-isotope thermometry has increasingly been used to reconstruct absolute temperatures in the geological record, based on measurements of the abundance of ^13^C–^18^O bonds in lattices of carbonate minerals, expressed as Δ_47_ (9). An advantage compared to conventional stable isotope analysis is that clumped-isotope-based temperatures are independent of the δ^18^O and δ^13^C value of the water from which a carbonate precipitated (9), and therefore allowing a wide range of applications in Earth sciences. Besides its common use in paleoclimatology to determine temperature changes in Earth surface and ocean environments (10–12) and in continental tectonics to reconstruct the thermal history of paleofluids and diagenetic minerals (13), clumped-isotope analyses have recently also shown great potential in constraining temperatures of carbonate phases involved in impact ejecta processes (14).

**Fig. 1.**
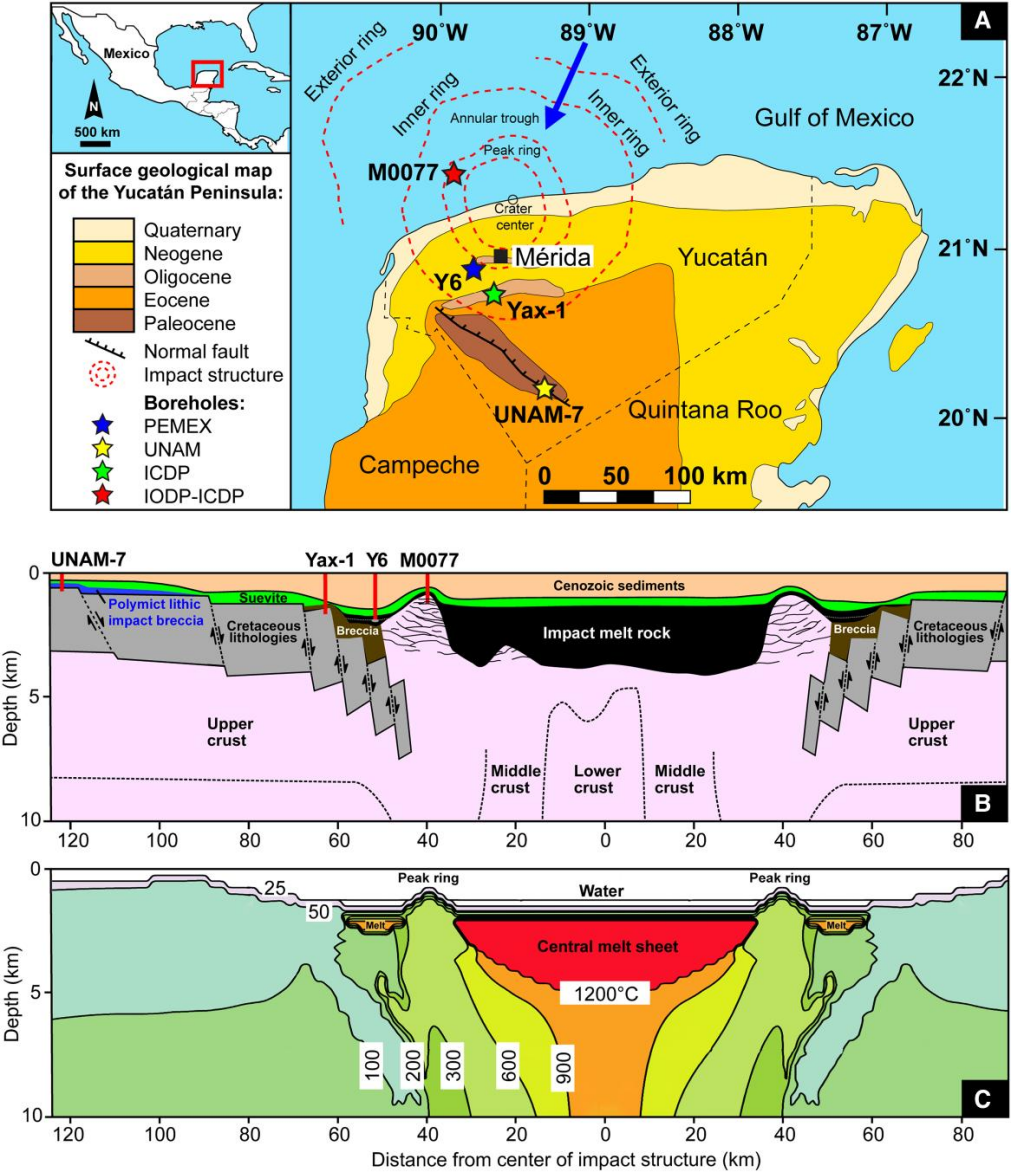
Chicxulub impact structure and sample locations. A) Simplified surface geological map of the northern part of the Yucatán Peninsula in México showing the location of the buried Chicxulub peak ring crater and the boreholes selected in this study (modified from Refs. [3, 4, 18–20]). The blue arrow indicates a gap in the inner ring of the Chicxulub structure and shows a potential pathway of water re-entering the crater after formation (4). B) Schematic geological cross-section through the Chicxulub impact structure displaying the boreholes and the interpreted sequence of crustal rock, Cretaceous carbonate basement (in gray), impact melt rock (in black), suevite (in green), breccia (Bunte breccia type in brown), polymict lithic impact breccia (in blue), and postimpact sediments (in orange, modified from Refs. [17, 20, 21]). C) Model of the Chicxulub postimpact hydrothermal system in cross-section, with isotherms representing the initial temperature distribution immediately after impact (modified from Ref. [22]).

The Chicxulub cratering event represents an ideal case study to apply the clumped-isotope method on the impactite record. The ∼12-km-sized Chicxulub asteroid impacted with a steep angle (45–60° to horizontal) (15) on a mixed crystalline-sedimentary target below the Yucatán Peninsula, composed of a ∼3-km thick Mesozoic carbonate-evaporite platform covered by a few hundred meters of water and situated on top of pre-Mesozoic granitic basement (16, 17) (Fig. 1). Carbonate components are present in the proximal Chicxulub impactites as fragmented sedimentary material in the form of coarse clasts and fine-grained particles in the matrix, as molten material in suevites (impact melt-bearing breccias) and impact melt rock, and as secondary phases precipitating in fractures (18, 21, 23, 24). However, the role and fate of these carbonates in the impact processes related to the Chicxulub cratering event, such as shock-melting, devolatilization, ejecta production, and potential contribution to postimpact carbon cycle perturbations, remain poorly constrained (25). Recently, Burtt et al. (14) performed conventional (δ^18^O–δ^13^C) and clumped stable isotope analyses on Chicxulub accretionary lapilli, which are rounded aggregates of carbonate and minor silicic glass as part of the proximal impact ejecta. These carbonate accretionary lapilli are derived from the Cretaceous–Paleogene (K–Pg) boundary site along the Brazos River in central Texas, at a paleodistance of ∼1,250 km from the center of the Chicxulub crater (26). These particles showed high clumped isotope-derived temperatures in excess of 300°C and are interpreted to have been formed through hot atmospheric processes linked to the Chicxulub impact vapor plume (14).

Clumped and conventional stable isotope analysis on impactites and carbonate target rocks within the Chicxulub impact structure offers a unique opportunity to unravel the complex thermal and material pathways from the source area to the global K–Pg impact ejecta layer (27). As clumped isotopes are independent of the isotopic composition of the fluid, this technique is not only appropriate for quantifying absolute formational temperatures of carbonate minerals but also for constraining the effect of a postimpact hydrothermal system where the fluids are likely highly variable in composition (28). The combination of impact heating and shock deformation generated a porous and permeable structure across the Chicxulub basin that was rapidly filled with ocean water following the impact (18, 29). Thermal modeling of this system suggests crater-wide hydrothermal activity for 1.5 to 2.3 Myr, with temperatures exceeding 1,200°C in the central melt sheet and 300°C in the peak ring region immediately after impact (22,28) (Fig. 1C). Testing this model is a main objective of the 2016 Expedition 364 drilling from the International Ocean Discovery Program (IODP) and International Continental Scientific Drilling Program (ICDP), which sampled the offshore part of the Chicxulub peak ring at site M0077 (Fig. 1A and B). Besides mineralogic and paleomagnetic data (28), clumped-isotope data from the uppermost part of the IODP–ICDP Exp. 364 impactite sequence have been used to confirm that temperatures exceeded ∼70°C in the seiche layers deposited within the first months to years after impact (30). However, temperature constraints farther down this drill core and in other Yucatán cores are needed to better understand the complexities of the Chicxulub impact cratering process, such as the spatial distribution and evolution of impact generated melt volumes and its associated hydrothermal system.

The microcrystalline calcite (micrite) present in the seiche layers in the IODP–ICDP Exp. 364 core (31) is thought to have formed by decarbonation of the Chicxulub target limestone, followed by rapid reprecipitation from the water column (30). This exothermic back-reaction of the decomposition products CaO and CO_2_ into CaCO_3_ has previously been suggested to explain micrite intervals of proximal marine K–Pg sites in the Gulf of México region (27). During the decarbonation process, degassing takes place that releases CO_2_ and H_2_O, and several studies simulated this process and measured the volatile release in a laboratory setting using shock experiments (32–34). However, extrapolating the amount of volatile release from a laboratory experiment to the entire Chicxulub crater has proven to be difficult based on limited studies on the Yucatán carbonate target lithologies (35). This hampers a precise quantification of the volume of impact-generated dust and climate-active gases, such as CO_2_ and SO*_x_*, released into the atmosphere following impact (6, 36). Such constraints are crucial to better determine the role of, e.g. impact dust and CO_2_ in the initial climatic and environmental stress after the Chicxulub impact (including the K–Pg impact winter [37]) and the long-term postimpact warming that lagged the K–Pg boundary mass extinction (26, 38, 39). Here, we present stable oxygen (δ^18^O), carbon (δ^13^C), and clumped-isotope (Δ_47_) data, accompanied by petrographic observations, for carbonate-bearing phases of impactites deep within and proximal (<50 km from the crater rim) to the Chicxulub impact structure (Fig. 1A and B), to further unravel the complex thermodynamic processes taking place in the aftermath of the K–Pg impact event.

## Results

**Fig. 2.**
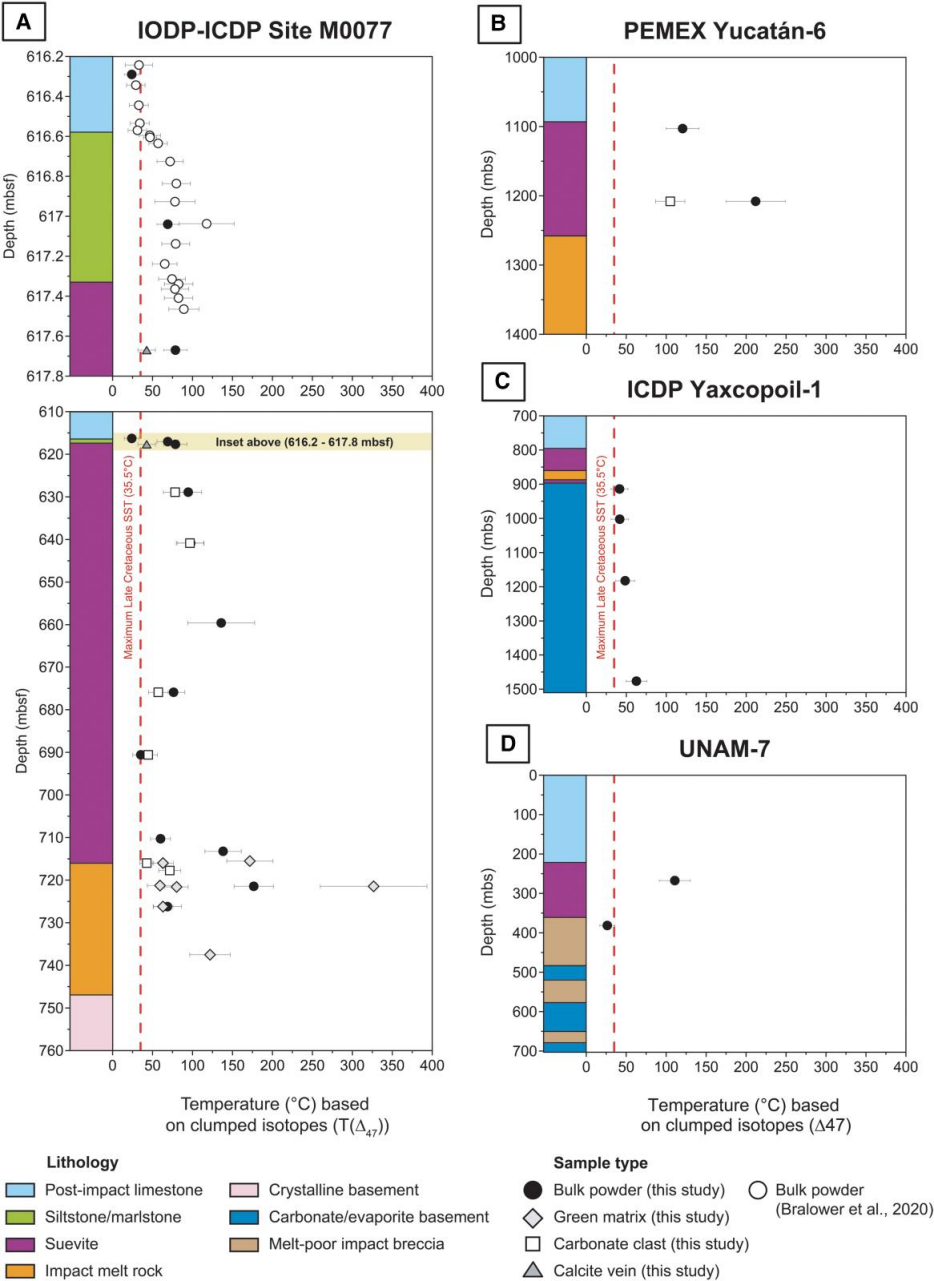
Stratigraphic overview showing *T*(Δ_47_) data vs. depth, lithology, and sample type of the four Chicxulub drill cores. A) IODP–ICDP Exp. 364 drill core from Site M0077, with in the inset between 616.2 and 617.8 mbsf also compiled *T*(Δ_47_) data from Bralower et al. (30); B) PEMEX Yucatán-6 (Y6); C) ICDP Yaxcopoil-1 (Yax-1); D) UNAM-7. The red dashed line represents the maximum Late Cretaceous SSTs, as compiled by Upchurch et al. (40). Error bars correspond to 2 SE.

The clumped isotope-derived temperatures (*T*(Δ_47_)) from carbonate phases in 36 samples from four different Yucatán drill cores (including impact [melt-bearing] breccias, impact melt rock and carbonate [target] rock from IODP–ICDP Site M0077, PEMEX Yucatán 6 [Y6], ICDP Yaxcopoil-1 [Yax-1], and UNAM-7; Figs. 1A, B and S1–S7; see details in Materials and methods) show a clear pattern when plotted in stratigraphic context and according to lithologic units (Fig. 2). Firstly, the large majority of the data yield temperatures in excess of 35.5°C, which represents the maximum Late Cretaceous sea surface temperature (SST), as compiled based on both proxy data and model simulations (40). Only two lithological units generate average temperatures lower than 35.5°C. These are the postimpact Paleogene limestones of the IODP–ICDP Exp. 364 drill core at depths above 616.58 meters below sea floor (mbsf; see inset of Fig. 2A), which provide an average *T*(Δ_47_) of 30.7 ± 6.1°C (1 standard error [SE]). A low *T*(Δ_47_) of 26.3 ± 4.7°C (1 SE) is also obtained for a sample from the melt-poor impact breccia interval of the UNAM-7 drill core outside of the crater (at 381.40 meters below the surface [mbs]; Fig. 2D).

Secondly, differentiating *T*(Δ_47_) data from bulk powders vs. specific carbonate phases indicates that in most cases, bulk powders yield higher temperatures than carbonate clasts or calcite veins from the same sample (Fig. 2A and B). An exception to this is the green matrix of the IODP–ICDP Exp. 364 brecciated impact melt rock (at a depth of 721.45 mbsf), which records the highest *T*(Δ_47_) of 327°C ± 33°C (1 SE) of this dataset. Thirdly, an overall increase in *T*(Δ_47_) values with depth is visible in the Y6 and Yax-1 core, with *T*(Δ_47_) increasing to 212 ± 19°C (1 SE) and 62.6 ± 6.5°C (1 SE), respectively (Fig. 2B and C). In the IODP–ICDP Exp. 364 core, the *T*(Δ_47_) pattern is more variable with values within the layered suevite (previously termed bedded suevite in (18)) and graded suevite ranging from ∼35 to 136°C. Values in the underlying non-graded suevite and impact melt rock interval vary between ∼43 and 327°C. Downwards from 713.23 mbsf, 5 out of 12 samples record a temperature in excess of 120°C. In contrast to the other three drill cores, the UNAM-7 drill core shows an opposite pattern (Fig. 2D), with the melt-rich suevite characterized by a much higher temperature than the underlying melt-poor impact breccia (111 ± 10°C vs. 26.3 ± 4.7°C; 1 SE).

**Fig. 3.**
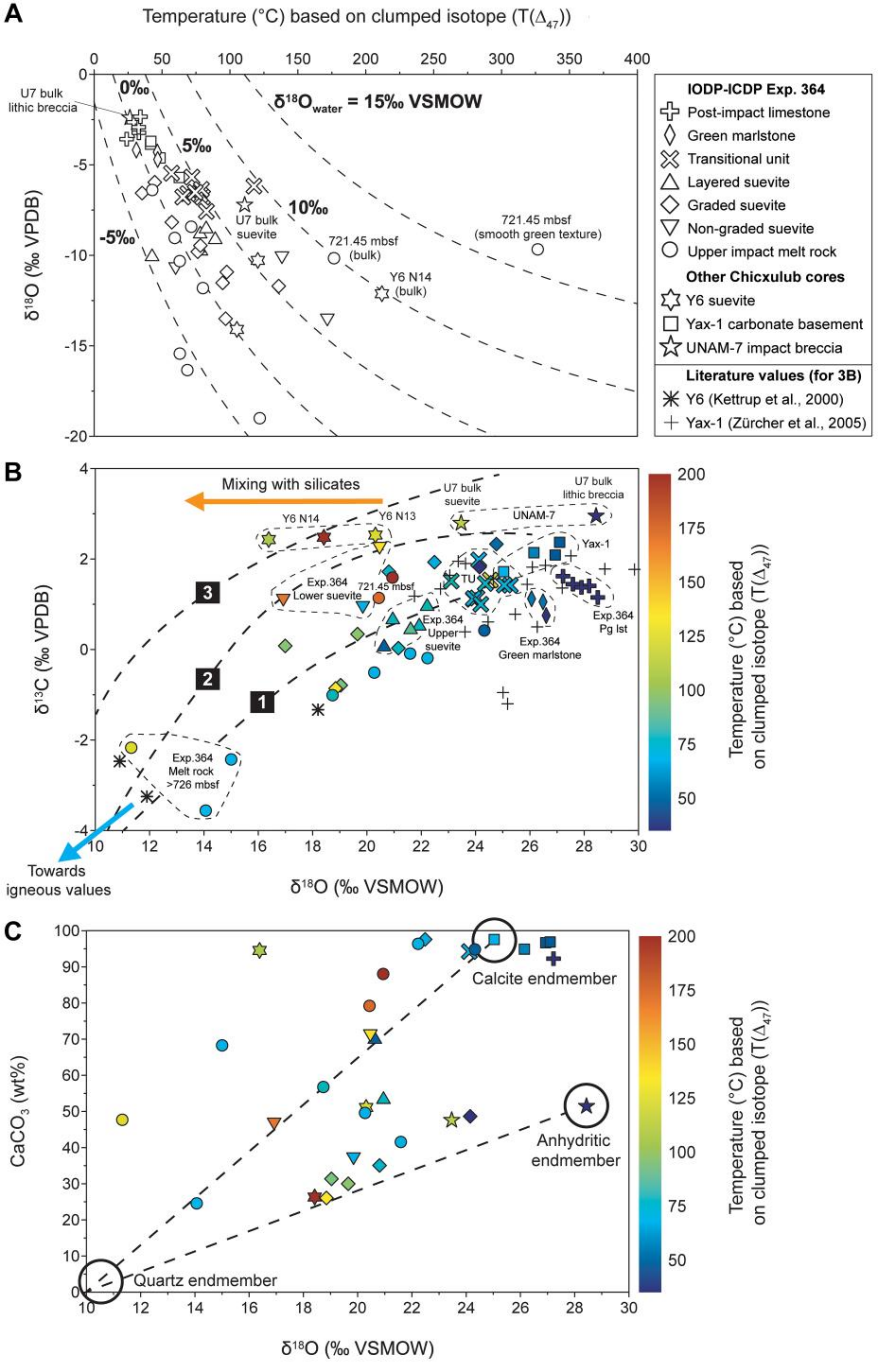
Isotopic cross-relationships of the Chicxulub impactites. A) Relationship between δ^18^O data and average *T*(Δ_47_) data with the symbols referring to the lithological units from the four different drill cores. Dashed lines are constant δ^18^O_water_ (VSMOW) values of possible diagenetic fluids based on the carbonate–water equilibrium relationships of O’Neil et al. (41). Data plotting along these curves would indicate alteration via secondary processes, which can explain a large part of the dataset but not all data points (see explanation in the main text). B) Isotopic cross plot showing δ^13^C vs. δ^18^O data, with colors indicative of the corresponding average *T*(Δ_47_) values. Dashed lines from right to left correspond to O-C isotope depletion patterns observed in contact metamorphic case studies 1 (Mount Royal pluton, Quebec), 2 (Alta Stock, Utah), and 3 (Pine Creek, California) as summarized in Baumgartner and Valley (42), reflecting a trend from unaltered carbonates in the upper right toward marbles and eventually igneous compositions in the lower left. Most of the isotopic signatures of the Chicxulub impactites from this study can be explained by hydrothermal processes similar to contact metamorphism. As a comparison, literature δ^13^C and δ^18^O values of Y6 (cores N15–N19) (43) and Yax-1 (between depths 800 and 883 mbs) (19) are shown (lacking clumped-isotope data) that largely follow the same trend. However, an IODP–ICDP Exp. 364 impact melt rock sample (721.45 mbsf) and suevites from UNAM-7, Y6, and IODP–ICDP Exp. 364 yield different isotopic compositions and higher *T*(Δ_47_), which likely reflects mixing with (molten) silicates, and the preservation of a signal primarily linked to thermal impact processes superimposed by the hydrothermal overprint. C) Relationship between δ^18^O and CaCO_3_ content (in wt%, based on µXRF analysis) of the different Chicxulub crater lithologies, with colors indicating the *T*(Δ_47_) data. The dashed lines represent the evolution of the δ^18^O composition of a marine carbonate (with typical unaltered calcite values around 25‰ VSMOW [44]) or an anhydrite-dominated lithology (exemplified by the UNAM-7 lithic breccia with ∼50 wt% CaCO_3_ and an δ^18^O value of ∼28‰ VSMOW) toward a quartz composition (with 0 wt% CaCO_3_ and an δ^18^O value of ∼10‰ VSMOW [44]). Large parts of the impactite dataset follow one of these lines, which illustrates the importance of mixing with silicate components. Exp. 364 Pg lst—Paleogene limestone of the IODP–ICDP Exp. 364 core (616.24–616.56 mbsf). TU—Transitional unit of the IODP–ICDP Exp. 364 core (616.56–617.34 mbsf).

The conventional stable isotope data (Figs. S8 and S9) display a stratigraphic pattern that mimics the trends of the *T*(Δ_47_) curves. The Paleogene limestones at the top of the IODP–ICDP Exp. 364 core show the least negative δ^18^O values, which is followed by a general decreasing δ^18^O trend into the layered suevite sequence (Fig. S8A). The graded suevite sequence displays an increasing trend below 660 mbsf, followed by the non-graded suevite and impact melt rock interval that shows a decreasing downward trend with δ^18^O values down to −19‰ Vienna Peedee belemnite (VPDB) or ∼11‰ Vienna Standard Mean Ocean Water (VSMOW). The Y6 and Yax-1 cores also display decreasing downward trends (Fig. S8B and C), in contrast to the increase with depth in UNAM-7 (Fig. S3D). The δ^13^C data (Fig. S9) display a pattern highly comparable to the δ^18^O curve, with low δ^13^C values down to −3.6‰ (VPDB) in the IODP–ICDP Exp. 364 impact melt rock interval (Fig. S9A), but an overall more modest downward trend in the Y6, Yax-1, and UNAM-7 cores (Fig. S9B–D). No relationship is observed between δ^13^C and *T*(Δ_47_) (Fig. S10), as also documented for K–Pg accretionary lapilli (14). In contrast, δ^18^O shows a weak correlation with *T*(Δ_47_) because most of the high *T*(Δ_47_) data fall in lower δ^18^O fields (Fig. 3A). Most samples plot along lines of constant δ^18^O_water_ (following O’Neil et al. [41]), indicating that diagenetic fluids played an important role in the δ^18^O_carbonate_ trends (14). However, several samples deviate from these δ^18^O_water_ isolines, including the UNAM-7 breccias, Y6 suevites, and a selection of IODP–ICDP Exp. 364 impact melt rock and lower suevite samples (Fig. 3A). This isotopic pattern might be linked directly to impact processes, as in general, volatilized CO_2_ has distinct ^13^C and ^18^O abundances compared to the starting carbonate (45), for which genetic models will be explored in the following sections.

## Discussion

### Ground-truthing a widespread hydrothermal system

The uppermost carbonates (earliest Danian in age) from the IODP–ICDP Exp. 364 core and the lowermost impact breccia from the UNAM-7 core are the only studied lithologies that display *T*(Δ_47_) < 35.5°C (Fig. 2). This trend suggests that the other lithological units have experienced, recorded, and preserved elevated temperatures that are not primarily linked to precipitation in near-equilibrium with the SST conditions of the Late Cretaceous ocean (40). The pre-impact carbonates present in the drill cores have been dated from Cenomanian to Maastrichtian ages (∼100–66 Ma), based on the micropaleontology of carbonate clasts in the IODP–ICDP Exp. 364 suevites (46) and based on Sr isotope stratigraphy of Yax-1 limestones (35). A low-latitude compilation of Cretaceous SST data based on TEX_86_ and planktonic foraminiferal δ^18^O constrains an SST evolution of ∼35°C around the Cenomanian–Turonian thermal maximum down to ∼25°C in the Late Maastrichtian (47). The Yax-1 limestone basement, dated to this period, displays *T*(Δ_47_) values in the range of 41.5–62.6°C, significantly higher than the expected paleoclimatic range. This implies the occurrence of widespread post-depositional alteration, which can also be inferred from petrography as Yax-1 microfossils within biomicritic limestones are still recognizable, but poorly identifiable (35) (Fig. S6C).

Clumped-isotope compositions of especially fine-grained matrix limestones and dolostones are susceptible to recrystallization and thermal resetting by diagenesis linked to, for instance, shallow burial conditions (48). *T*(Δ_47_) values of fine-grained carbonates from a deep drill core from the Bahamas showed that at 1.3 km depth a sudden increase by 10°C occurred without distinct changes in petrologic texture or conventional stable isotope values (48). Hence, *T*(Δ_47_) values from shallowly buried carbonates suggest that fine-grained samples should be used with extreme caution in clumped-isotope studies seeking primary environmental temperatures. In the IODP–ICDP Exp. 364 drill core, the borehole-fluid temperature increases progressively downhole from ∼26 to 66.5°C at the base of the hole (1,334.69 mbsf). Within the studied interval (until 737.5 mbsf), the borehole temperatures reach 47.5°C (49). The majority of the *T*(Δ_47_) values of the IODP–ICDP Exp. 364 suevite and impact melt rock intervals (Fig. 2A) are above this 47.5°C value, so the geothermal gradient alone cannot explain the variations seen in the clumped isotope data. In contrast to the Bahamas case study (48), we also observe a clear change in δ^18^O and δ^13^C values with depth vs. the non-altered values from the uppermost carbonates and lowermost UNAM-7 impact breccia. We suggest that a mixture of burial diagenesis with a hydrothermal overprint can explain the majority of the observed variations. In this case, graded suevite sample 54_1_64_66 (659.58 mbsf) can be used as an example as it yields a high *T*(Δ_47_) value of 135.6 ± 20.9°C (1 SE). This sample is positioned close to a red-orange zone that has been interpreted by Kring et al. (28) as a hydrothermal channel (Fig. S2G). The carbonate matrix of this suevite was likely dissolved in the hydrothermal channel, before Na-dachiardite, analcime, clay, and sparry calcite partially filled the channel (28). The *T*(Δ_47_) value of such a suevite bulk powder therefore reflects a partial temperature overprint by hydrothermal processes that can be heterogeneous throughout the drill core stratigraphy.

To further disentangle the observed *T*(Δ_47_) pattern at Chicxulub, we generated an isotopic crossplot (Fig. 3B) in which the δ^18^O vs. δ^13^C signatures are linked to specific processes related to variable shock heating conditions, different carbon sources, and/or post-depositional alteration (14). In general, the region with the most positive δ^18^O and δ^13^C values is related to the lowest *T*(Δ_47_) values. A clear trend is visible from this region toward very low δ^18^O and δ^13^C values, which are interpreted to result from a secondary hydrothermal overprint. This trend is largely consolidated with depth in the IODP–ICDP Exp. 364 core since offsets are visible between the Transitional unit and the layered suevite toward impact melt rock intervals at a depth of >726 mbsf (Fig. 3B). This pattern shows strong similarities with O-C isotopic trends found along transects in contact metamorphic aureoles (42), as exemplified here with the well-studied Mount Royal pluton in Quebec, Canada (50). In this case study (see line 1 in Fig.3,B; summarized in Baumgartner and Valley [42]), the unaltered Trenton marine limestone (with an average δ^18^O and δ^13^C of 24.1‰ VSMOW and 1.1‰ VPDB, respectively) is progressively changing into a marble and finally into calcite phases present within syenite and gabbro from an alkalic intrusion, the latter yielding average δ^18^O values of 10.1‰ VSMOW and δ^13^C values of −4.9‰ VPDB. These values also match stable isotopic data from carbonatite subvolcanic intrusions from the Miocene Kaiserstuhl alkaline complex in southwestern Germany, with average values for δ^18^O of 7.8‰ VSMOW and for δ^13^C of −5.5% VPDB (51). This trend toward igneous values is also observed downcore in the IODP–ICDP Exp. 364 core with the lowest δ^18^O and δ^13^C values (down to 11‰ VSMOW and −3.6‰ VPDB, respectively) present in the impact melt rock samples from 726 mbsf downwards. Published δ^18^O and δ^13^C values of the impact melt rocks from Y6 (43) and the suevites and impact melt rocks from the Yax-1 drill core (19) also follow the depletion line of the Mount Royal case study (Fig. 3B). An exception to this is a small selection of impact melt rock samples with (very) negative δ^13^C values attributed to the influence of hydrocarbon-bearing target rocks (19), a pattern that is not observed in the IODP–ICDP Exp. 364 core.

The IODP–ICDP Exp. 364 samples below 726 mbsf are characterized by the presence of zones of green schlieren (Fig. S1, samples #7–8), which consist of Fe-Mg rich phyllosilicates (∼60%, mostly saponite) mixed with calcite (∼25%), garnet (≤15%), and minor opaque minerals (18, 21, 24, 52) (Fig. S5). The presence of hydrothermal andradite garnet with grossular-rich rims within the fluid-filled cavities of the green schlieren zones suggests precipitation from hydrothermal fluids with minimum temperatures of ∼280 to 300°C (28, 53). Saponite formed at ∼100 to 150°C based on paragenetic sequence analysis in the Ries crater (54), which roughly match the *T*(Δ_47_) data determined for the saponite dominated melt rock samples below 726 mbsf (63–122°C). Recent isotopic analysis (δ^18^O and δ^2^H) of the clay-separated fraction of the IODP–ICDP Exp. 364 suevites and melt rocks reveal a low temperature (∼20 to 50°C) and meteoric-water dominated origin for the Chicxulub smectitic clays, including saponite (55). Therefore, as the coarser grained suevites and clast-rich impact melt rocks in our sample set consist of a mixture of carbonate and non-carbonate components (including clays, quartz, and garnet), whole-rock analyses and analyses of green schlieren phases likely represent a mixture of isotopic signals, explaining the large variability in *T*(Δ_47_).

Nevertheless, the *T*(Δ_47_) data agree with numerical thermal models of the Chicxulub impact structure and our results confirm a crater-wide effect of the hydrothermal system on the impact and target lithologies (22, 28). The Yax-1 samples, situated in the terrace zone, yield *T*(Δ_47_) values around 50°C, as predicted by the model (Fig. 1C). The Y6 samples, positioned within the annular trough on top of a predicted melt pool, show *T*(Δ_47_) values >100°C for suevite samples from the upper impactite sequence. Impact melt rock from IODP–ICDP Exp. 364 Site M0077, located on top of the granitic peak ring, even yield a *T*(Δ_47_) > 300°C, which is largely in line with the thermal model of Abramov and Kring (22), although these values are expected at slightly greater depths. The porous peak ring zone is especially susceptible to hot fluid flow from the neighboring central melt sheet (28). However, the effects of the distance from the melt sheet and its cooling history on the general temperature and fluid flow throughout the Chicxulub crater are not well-constrained. Hence, clumped-isotope data on a wider suite of proximal impactites throughout an impact structure provide new quantitative insights in the thermal and spatial evolution of impact-generated melt sheets and hydrothermal cells, which aid in identifying suitable habitats for microbial ecosystems to form as impact basins cool (28).

### Implications for impact ejecta processes

Besides the hydrothermal overprint, we found evidence of an additional isotopic signature in the Chicxulub impactites that cannot be explained by hydrothermal effects only (Fig. 3). This is exemplified by the UNAM-7 samples located outside the exterior rim of the Chicxulub crater (Fig. 1A), in a region which is characterized by no or a very minimal hydrothermal overprint. This is based on a petrographic assessment revealing, e.g. well-preserved foraminifera (Fig. S7), and based on non-traditional isotopic analysis of the UNAM-7 suevite showing Fe, Cu, and Zn isotope ratios all plotting in the Upper Continental Crust range (56). Diagenetic fluids cannot explain the *T*(Δ_47_) increase of ∼85°C and the ∼5‰ positive shift in δ^18^O between the UNAM-7 lithic breccia and overlying suevite (Figs. 2D and S8D), as both samples do not plot on the same carbonate–water equilibrium curve (Fig. 3A). To fully understand the deposition of the lithic breccia and suevite unit in the UNAM-7 drill core, it is important to envision the different impact-generated processes that are at play just outside of the exterior rim of the Chicxulub crater in the direct aftermath of the impact event.

At the UNAM-7 locality, situated on the proximal ejecta blanket 126 km SE of the center of the Chicxulub crater (Fig. 1A and B), the first impact-generated process that affected this location was most likely the outgoing pressure wave that was initiated by the Chicxulub impact event. Due to this massive shock wave, extensive earthquakes occurred that caused the Yucatán and Gulf of Mexico carbonate platform to collapse (57). This triggered, within one to a few minutes after impact, large-scale brecciation of the sedimentary target rocks. This type of brecciation has been observed at, for instance, the K–Pg boundary site at El Guayal, ∼520 km SW of the Chicxulub crater center, where a ∼45 m thick carbonate-rich lithic megabreccia sequence was found (58). We envision such a seismic-induced brecciation process also at the UNAM-7 locality, where a lithic breccia sequence of ∼320 m thick was encountered (Fig. 2D) (59). The studied polymict lithic breccia sample at 381.4 mbs contains carbonate and anhydrite clasts while lacking (abundant) silicate impact melt particles and shocked target rock material (Fig. S7B). It yields a *T*(Δ_47_) of 26.3 ± 4.7°C (1 SE) and we therefore interpret it as a carbonate-rich sedimentary rock that was initially formed in near-equilibrium with the SST conditions of the Late Cretaceous ocean. It became brecciated due to the shock-induced seismic activity without any additional heating process involved.

After the initial shock wave, proximal impact ejecta was deposited within several minutes outside of the Chicxulub crater exterior rim in the form of a hot ejecta curtain containing a ground-hugging flow (59). Field evidence for such a ground-hugging flow has, for instance, been documented at the K–Pg section of El Ramonal at ∼340 km SE of the Chicxulub crater center. At that locality, the spheroid bed from the Albion Formation contains melt particles and carbonate rip-up clasts from the underlying Upper Cretaceous Barton Creek Formation, indicative of deposition through a hot flow with a strong erosive force (60). In addition, the Yucatán area was most likely subject to an outward-moving tsunami wave, induced by the shock wave of the Chicxulub impact. Evidence for sedimentation caused by such a rim wave tsunami has been found in drill cores and outcrops all around the Gulf of Mexico (57). The present-day Yucatán Peninsula was at ∼66 Ma a shallow shelf with water depths <50 m south of the impact site and ranging from 100 to 2,000 m within the area impacted (a carbonate ramp) (4, 61), so it was a likely suitable region for generating a rim wave tsunami. The suevite unit at UNAM 7 contains silicate impact melt particles and shocked target rock material (Fig. S7A), including petrographic features that hint toward shock-induced anhydrite decomposition and recrystallization (59). This hints toward a high-temperature depositional process for the Chicxulub outer suevite. However, the *T*(Δ_47_) value of the UNAM-7 suevite (111 ± 10°C), measured on a bulk powder, is not as high as expected from a pure ground-hugging flow signature. Therefore, we suggest that cooling took place from water derived from the impact-induced rim wave tsunami (57). This outgoing tsunami most likely reached the UNAM-7 locality at the same time or slightly after the ground-hugging flow passed over (57), thereby likely overprinting the *T*(Δ_47_) value and causing a mixed, overall cooler signal.

The results of Burtt et al. (14), which focused on K–Pg impact ejecta from the Brazos K–Pg site, are interesting to further interpret the *T*(Δ_47_) and isotopic records at Chicxulub. However, this study relied on a different standardization, without including ETH (Eidgenössische Technische Hochschule Zürich) standards that are used in the universally accepted I-CDES (intercarb-carbon dioxide equilibrium scale) reference frame (62). Since 2021, this framework has been used within the clumped-isotope community for valid result comparison. Consequently, it is not possible to fully recalculate the absolute temperatures of Burtt et al. (14). Even though the comparison remains relative, it becomes apparent that both the UNAM-7 proximal ejecta record and the Brazos K–Pg ejecta section plot within similar *T*(Δ_47_) and δ^18^O fields (Fig. S11A). An analogous shift in δ^18^O compared to the UNAM-7 lithic breccia to suevite transition is observed at Brazos, when uppermost Cretaceous foraminifera and Paleogene carbonate mudstones (yielding δ^18^O values between −1.3 and −3.0‰ VPDB; interpreted to have been precipitated in near SST-equilibrium) are compared to unaltered accretionary lapilli (δ^18^O values between −5.4 and −7.7‰ VPDB) (14) (Fig. S11A). The associated *T*(Δ_47_) change is, on average, in the order of 80–120°C, although some lapilli yield very elevated *T*(Δ_47_) in excess of 300°C (14). These extreme temperatures in the accretionary lapilli are interpreted to be linked to heating from atmospheric reentry (63) or due to the hot conditions in the surficial thermal pulse within the first minutes after impact (14). The observed δ^18^O and δ^13^C trends in these accretionary lapilli (Fig. S11B) can be explained by Rayleigh distillation of ^18^O and ^13^C from the Chicxulub carbonates, representing fractionation during impact decarbonation linked to the vapor-rich impact cloud (14).

At the UNAM-7 site, no accretionary lapilli have been found. Their clumped and conventional stable isotopic signatures are based on a bulk suevite powder resulting in a mixture of different components (silicate melt, anhydrite, and carbonate clasts in a fine-grained clastic matrix; Fig. S7A) explaining the generally lower *T*(Δ_47_) compared to measuring specific ejecta components. The δ^13^C values in our Chicxulub dataset are also significantly higher relative to those of the accretionary lapilli (+3‰ vs. −3 to −10‰ VPDB, respectively), plotting above the Rayleigh distillation curves (Fig. S11B). This implies for UNAM-7 that thermal decarbonation following atmospheric fractionation processes is not a main factor in the Chicxulub outer suevite deposition and other mechanisms are more dominant such as mixing with silicate components (42, 45) during proximal ejecta density current processes (64) and/or tsunamigenic processes (57). Quantifying the formational temperatures of (bulk or isolated) carbonate ejecta in the Yucatán proximal ejecta blanket elucidates the complex dynamics of melting, mixing, volatilization, and atmospheric interaction in the Chicxulub pyroclastic-like density currents, ejecta curtain, and/or vapor plume (63). Moreover, it can also place constraints on emplacement scenarios for lithic impact breccias and suevites from other impact structures with a sedimentary carbonate target, such as Haughton (45, 65), Ries (16, 64, 66–68), Barringer (69), and potentially on impact structures on Mars such as Leighton crater and the Huygens impact basin (70).

### Formation of hot within-crater carbonate phases

A large fraction of the dataset from within the Chicxulub crater, including Yax-1 and the IODP–ICDP Exp. 364 transitional unit, upper suevite, and the majority of the graded suevite and impact melt rock, follows the δ^18^O–δ^13^C line of contact metamorphic case study 1 (42) (Fig. 3B). However, 7 out of the 10 samples that yield *T*(Δ_47_) > 100°C, do not plot on this line as these are situated in a restricted field with low δ^18^O values ranging between ∼16 and 21‰ VSMOW and positive δ^13^C values varying between ∼2.5 and 1‰ VPDB. This field could be partly explained by the pattern seen in other contact metamorphic case studies, as exemplified by the dolomites from the granodioritic Alta Stock metamorphic aureole in Utah (71) (#2 in Fig. 3B) and the calc-silicate skarn of Pine Creek, California (72) (#3 in Fig. 3B). However, the intrusion of granitic magma within the upper continental crust takes place in the order of millions or tens of millions of years (73). Therefore, the initial heating process of long-lived contact metamorphism is strikingly different compared to geologically instantaneous impact heating at Chicxulub, which likely prevents reaching thermodynamic isotopic equilibrium (45). The narrow range in δ^18^O and especially δ^13^C of these seven Chicxulub samples (Fig. 3B) probably indicates that these samples experienced only early diagenesis, as the δ^18^O values of successively deposited carbonate become increasingly more negative while the δ^13^C remain nearly constant during early diagenesis (74). As such, we expect the effects of the hydrothermal fluids on these high *T*(Δ_47_) values to be rather limited.

The generally low δ^18^O values can likely be explained by high-temperature reactions between molten silicate and carbonate phases, in which the silicates act as a reservoir of oxygen, resulting in fractionation to lighter δ^18^O values in the carbonate phase (45). This can be deduced by plotting the δ^18^O composition vs. CaCO_3_ concentrations (Fig. 3C), which shows the evolution of an unaltered marine carbonate (with a typical value of ∼25‰ VSMOW [44]) after equilibration with silicates (using ∼10‰ VSMOW a typical value for quartz [44]) at 1,200°C, if oxygen isotope exchange is complete (45). Our data roughly follow this carbonate–silicate mixing line and deviations can likely be explained by changing the δ^18^O values of the starting material, for instance, by considering evaporitic target rock as exemplified by the UNAM-7 lithic breccia (yielding ∼50% calcite, ∼50% anhydrite, and a δ^18^O of ∼28‰ VSMOW; Figs. 3C and S1). The isotopic exchange between carbonate and silicate phases during impact events has also been petrologically corroborated by Martinez et al. (45), who found δ^18^O values between 15 and 20‰ VSMOW in naturally shocked carbonates from the center of the Haughton impact structure in Canada. These naturally shocked carbonates are isotopically much lighter than unshocked carbonate target rock (δ^18^O of 23 to 29‰ VSMOW) and are also associated with the presence of Ca, Mg-enriched silicate glass, larnite (Ca_2_SiO_4_) and Ca-rich dendritic pyroxenoid (wollastonite, CaSiO_3_). Wollastonite is also found deep in the Y6 drill core in the Chicxulub crater as part as a reaction front between a carbonate fragment and a silicate melt matrix (25), potentially explaining the low δ^18^O values of the Y6 samples analyzed in this study (+16 to +20‰ VSMOW). The shocked carbonates from Haughton are associated with highly positive δ^13^C values up to even +9‰ VPDB (45). These samples show microstructures of secondary-formed carbonates, and it has been interpreted that these phases were formed as the result of absorbing large amounts of CO_2_ with a heavy δ^13^C signature, possibly recombined with residual CaO (lime) seconds after the impact (45). Most of our high *T*(Δ_47_) Chicxulub samples plot above ∼1‰ VPDB δ^13^C (Fig. S12), a value typical for unaltered marine limestones (44), and their positive δ^13^C values might be explained by similar devolatilization and recombination processes upon impact.

**Fig. 4.**
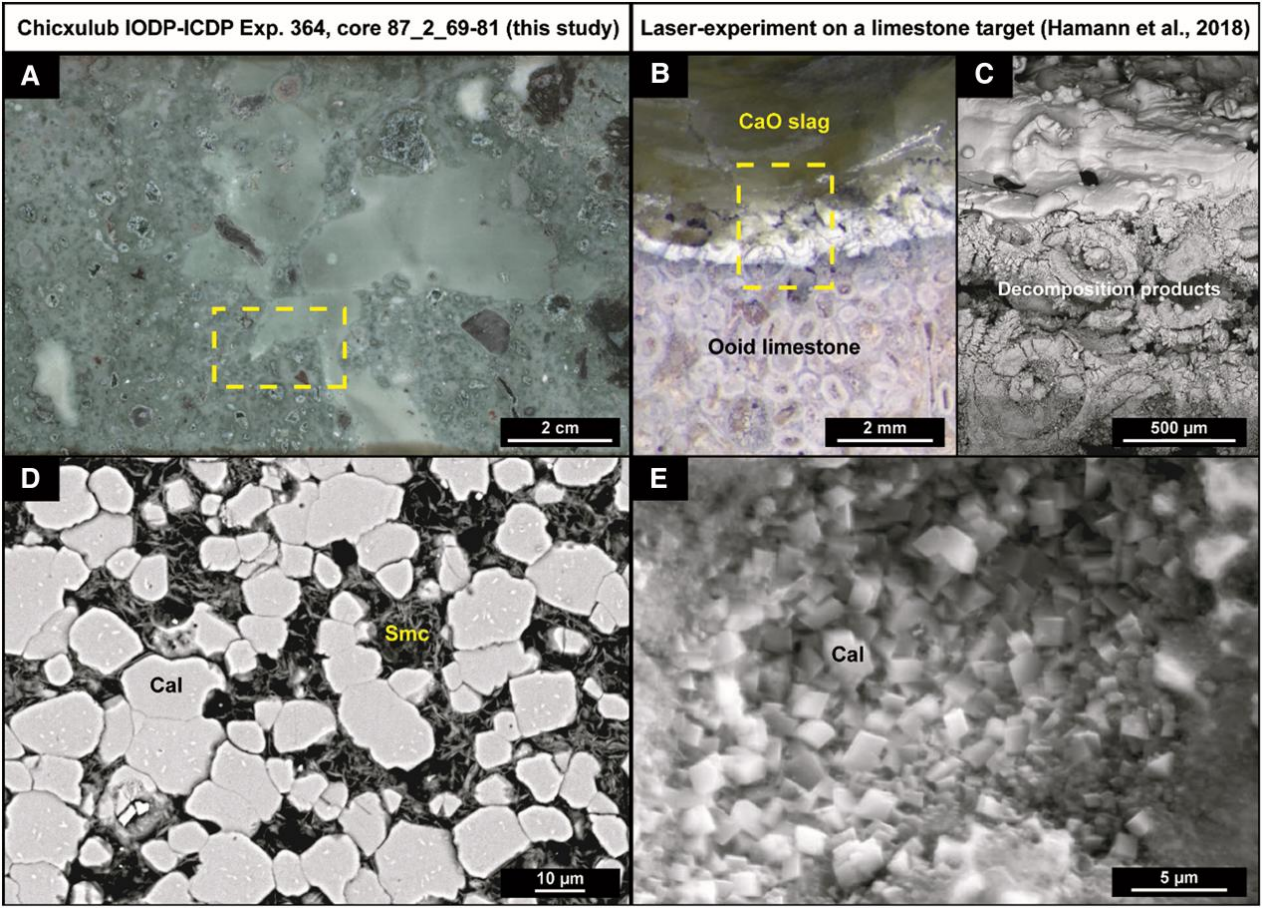
Petrographic similarities between Chicxulub samples and experimental analogs. The IODP–ICDP Exp. 364 impact melt rock interval, yielding high *T*(Δ_47_) (327 ± 33°C [1 SE]), shows similar macro- and micro-textures compared to results from laser-irradiation experiments on a limestone target (8). A) Halfcore photograph of IODP–ICDP Exp. 364 core section 87_2 (69–81 cm; 721.35–721.47 mbsf) displaying smooth green textures, which were analyzed in this isotopic study (see yellow area and Fig. S1B sample #6). B) Reflected-light photomicrograph and C) zoomed-in scanning electron microscopy-backscatter electron (SEM-BSE) image of an ooid-limestone plate after laser irradiation, showing successive thermal decomposition of CaCO_3_ resulting in white decomposition products and eventually in lustrous green CaO slag (modified from Hamann et al. [8]). D) Equigranular-like calcite matrix of the smooth textures in sample 721.45 mbsf, displaying subhedral calcite (Cal) grains of ∼5–15 μm in size, interspersed with minor smectite (Smc). E) Equigranular matrix of the zone with the decomposition products from the laser experiment showing euhedral calcite crystallites of ∼1–2 μm in size (modified from Hamann et al. [8]).

To further unravel the nature of these thermal impact processes, we focus on a petrographic comparison between experimental analogs and the IODP–ICDP Exp. 364 impact melt rock interval around 721.45 mbsf (Fig. 4), as this interval yields the highest *T*(Δ_47_) values of this study. The associated sample is situated in drill core 87R2 within a ∼30-cm thick blue–green zone (Fig. S1A), which displays enigmatic smooth green textures (Fig. 4A). Both bulk analysis and phase-specific analysis on the green textures have been performed, yielding *T*(Δ_47_) of 177°C ± 12°C (1 SE) and 327°C ± 33°C (1 SE), respectively. These two results do not follow a line of constant δ^18^O_water_ (Fig. 3A), suggesting that diagenetic fluids did not steer the isotopic signature. Interestingly, samples only ∼15 cm above and below 721.45 mbsf (Fig. S1A) do not exhibit this smooth texture and do not yield high *T*(Δ_47_) (59 ± 8°C and 80 ± 7°C, respectively [1 SE]). Therefore, the low *T*(Δ_47_) record there is likely governed by hydrothermal processes associated with the green schlieren phase. The smooth green texture at 721.45 mbsf strongly resembles dark green lustrous CaO slag that was experimentally produced through 1-bar laser melting of a basalt plate on top of an ooid-limestone plate (8) (Fig. 4B). A gradual increase in backscatter electron (BSE) brightness is visible along the laser transect (Fig. 4C), corresponding to an increase in Ca and decrease in O and C contents as measured by scanning electron microscopy-energy dispersive X-ray (SEM-EDX) analyses (8). This change is directly linked to decarbonation with the decomposition reaction following CaCO_3_ (s) → CaO (s, l) + CO_2_ (g), resulting in the presence of residual, highly reactive solid or liquid CaO (lime) (32). Impact melt rock sample 721.45 mbsf is petrographically characterized by microcrystalline silicate melt rock particles floating in an equigranular calcite matrix showing ∼5–15 μm subhedral calcite grains (Figs. 4D and S4). Although yielding a larger grain-size, this microtexture shows similarities to the equigranular matrix of 1- to 2-μm-sized euhedral calcite crystallites (Fig. 4E) that is found in the laser-heating experiment within the zone of decomposition products between intact calcite and CaO slag (8) (Fig. 4C). These calcite crystallites are interpreted as the products of rapid and in situ back-reactions of residual CaO and not yet discharged gaseous CO_2_, following the recombination reaction CaO (s, l) + CO_2_ (g) → CaCO_3_ (s) (32). Based on degassing and back-reaction experiments of calcite, Agrinier et al. (32) concluded that this residual CaO is highly reactive in the presence of CO_2_ after the initial postshock stage in the 300–700°C range, which matches the high *T*(Δ_47_) value recorded at 721.45 mbsf.

The minor textural differences (grain-size, grain-shape, and incorporation of phyllosilicates) between IODP–ICDP Exp. 364-based and experimental observations of this back-reaction (Fig. 4D and E) can largely be explained by a difference in timing and degree of post-depositional alteration. The small euhedral calcite crystallites (<2 μm) found in the laser experiments were produced within ∼40 s after irradiation, following quenching from >2,100 to ∼300°C (8). Similar but larger (<10 μm) textures produced in shock experiments took ∼200 s to form (32), which corresponds largely to the estimated timing of peak ring formation at Chicxulub and its main impact melt rock emplacement (21, 29). This duration of <5 min may also be a realistic estimate for the timeframe of the rapid formation of the secondary, subhedral calcite crystals (5–15 μm) in impact melt rock sample 721.45 mbsf (Figs. 4D and S4). As a snapshot of this process, the preserved high *T*(Δ_47_) signal may be the result of the recombination process of CaO and CO_2_, despite later subjection to hydrothermal smectite alteration. This means that the *T*(Δ_47_) of 327 ± 33°C (1 SE) likely represents an underestimation of the initially rapidly locked-in temperatures, which were in a later stage altered by cooler mixing processes. Garroni and Osinski (75) recently interpreted this ∼30 cm thick section as a flow calcite that represents remnants of hot impact-derived carbonate melt that was mixed with (colder) impact breccia material.

The overlying transition zone between impact melt rock and suevites (∼721–710 mbsf) in the IODP–ICDP Exp. 364 drill core is interpreted to have been formed due to a complex interplay of different impact-generated processes that occurred in the peak ring region within the first hour after impact (18, 24, 29). Chronologically, these processes likely started with outward flowing melt and hot ground-hugging density currents at the base. This was followed by melt-water interactions due to the first seawater ingress that occurred within the recently formed impact crater. We suggest that the non-graded suevite unit (715.6–710 mbsf) represents a mixture of these different impact processes since the *T*(Δ_47_) values show a decrease up the stratigraphy, from 171 ± 14°C (1 SE) to 60 ± 6°C (1 SE) ∼5 m above (Fig. 2A). We interpret that the base of the sequence was still hot, likely due to the high-temperature, melt-bearing density current processes, and the proximity to an impact melt body. This initial hot and rapid emplacement is corroborated by the fact that paleomagnetic measurements across the entire impact melt rock and non-graded suevite interval (∼760–710 mbsf) in the IODP–ICDP Exp. 364 drill core show consistent negative inclination values (28, 29). Additional evidence for a hot impact-related emplacement process for the non-graded suevite comes from the δ^18^O and δ^13^C values that plot in a field similar to the hot impact melt rock sample 721.45 mbsf (Fig. 3B). Moreover, petrographic analyses show that the non-graded suevite is dominated by angular vitric melt clasts and small (sub)angular recrystallized calcite grains in the matrix (Fig. S3). Partly recrystallized, but still taxonomically recognizable planktic foraminifera have also been found isolated in this clastic matrix (Fig. S3A) (18), which suggests that the temperatures were not high enough to fully recrystallize these marine microfossils. We therefore interpret that the non-graded suevite must have been cooled from above by ocean water flowing into the crater (18), which has been estimated to have occurred <30 min after impact (18, 29).

From 710 mbsf upwards in the IODP–ICDP Exp. 364 drill core an abrupt change is visible in (isotope) geochemical composition, petrography, and paleomagnetic signal, marking the onset of the deposition of the graded suevite unit. The CaO content is much lower than the underlying non-graded suevite (on average ∼20 vs. ∼40 wt%; Fig. S3C vs. Fig. S2F) (18) and a much wider range in δ^18^O and δ^13^C values is observed (Fig. 3B). Petrographically, the graded suevite shows a much larger variability in different target lithologies both in terms of clasts and matrix components (Fig. S2C–F). Magnetostratigraphy of the graded suevite sequence displays a random distribution with a wide diversity in both negative and positive inclination data (28, 29). We interpret that the graded suevite represents the full ocean resurge within the crater in the first hours after impact (18, 24, 29). In contrast to the initial sea water ingress in the crater, this ocean resurge was debris-laden and hence incorporated various target lithologies, with different initial paleomagnetic and temperature signatures, that were rapidly cooled due to mixing processes (18). We suggest that mixing with cold breccia material, superimposed on hydrothermal alteration, can explain the generally low *T*(Δ_47_) values (<100°C) of the graded suevite. We envision a similar scenario for explaining the temperature record of the overlying layered suevite, that also displays lower *T*(Δ_47_) values (<90°C). This unit most likely formed by seiche activity in the crater when the energy dissipated after the full ocean resurge, so mixing with colder breccia material and winnowing processes played an important role in its deposition (18, 31).

Finally, the IODP–ICDP Exp. 364 transitional unit is characterized by small (sub)angular calcite grains (Fig. S2B), which Bralower et al. (30) attributed to back-reaction processes as well. However, in contrast to the impact melt rock interval, this reaction would take place in close association with ocean waters, likely supersaturated in Ca and CO_2_ from degassed carbonates (27), when the crater region became flooded. This back-reaction probably took place in the months to years after the impact during suspension and settling in the water column or during early burial (30).

### CO_2_ release at the K–Pg boundary

The impactites in the Y6 drill core, located in the southern annular moat (Fig. 1), also experienced and recorded high primary temperatures as exemplified by *T*(Δ_47_) values between 105 and 212°C (±9–19°C [1 SE]) and isotopic trends in the upper and middle suevite not following a δ^18^O_water_ isoline (Fig. 3). Jones et al. (76) and Claeys et al. (23) found in the Y6 N13 upper suevite unit several distinctive feathery or spinifex textures in 1- to 3-mm-sized elongated, isolated calcite grains and in 30- to 300-µm-sized calcite grains in the fine clastic matrix (Fig. S6A and B). Based on comparisons with carbonatitic volcanic rocks and synthetic experiments, feathery calcite is interpreted to have quench crystal morphologies that formed through rapid crystallization from a carbonate-rich melt when this liquid admixed with cold breccia material (66, 76). It was estimated that up to 15% of all carbonate phases in Y6 represented molten material (23). The absence of feathery calcite in the other Chicxulub drill cores (Figs. S2–S7) and more positive δ^13^C values for Y6 compared to IODP–ICDP Exp. 364 (Fig. 3B) might indicate two different areas in the Chicxulub crater that are more susceptible to either impact melting (Y6 on the annular moat melt pond) or devolatilization (IODP–ICDP Exp. 364 from Site M0077 on the raised peak ring; Fig. 1C). This heterogeneous response can also be deduced from fragments of anhydrite that are still preserved in all Y6 suevite units (Figs. S1C and S6A), suggesting that devolatilization was less important in this crater region. On the other hand, the IODP–ICDP Exp. 364 core is devoid of anhydrites and other evaporites, implying intense shock vaporization of an extensive part of the evaporite (and potentially carbonate) target stratigraphy in the peak ring area (18, 29). Moreover, IODP–ICDP Exp. 364 upper impact melt rock samples have revealed light Cu and Zn isotopic signatures, superimposed on a hydrothermal signal, indicating a possible genetic link with distally ejected K–Pg material, and hinting also toward an enhanced (shock) volatilization and condensation in this unit (56). These independent data support our results, highlighting the potential of impact melt rock units in carbonate-bearing targets to record and preserve a signature linked to early thermal impact processes such as devolatilization. An interesting comparison can be made between Chicxulub and the ∼23-km-wide Haughton impact structure in Canada. Stable isotopic and petrographic work on Haughton impactites showed that the outgassing of carbonates is limited to a narrow zone in the center of this crater, where shock pressures reached 60 GPa (45). When extrapolated to Chicxulub, this would imply a central zone between 30 and 50 km that reached shock pressures sufficiently high enough for devolatilization (36).

Besides these crater heterogeneities, numerical modeling of volatile release during impacts should take into account the effect of recombination or back-reaction as this is likely a general mechanism for retaining volatile species after large collisions. Martinez et al. (45) and Agrinier et al. (32) showed that large fractions of the impact-produced CO_2_ from degassing of carbonate target rocks are lost due to rapid recombination with highly reactive CaO to CaCO_3_, leading to a reduction of CO_2_ injected in the atmosphere between 31–63 and 39–78%, respectively (at temperatures <700°C, with ambient pressures, and in a timeframe of <200 s). The most recent climate-impact modeling effort of Chicxulub considered full vaporization to occur and did not include these back-reactions in the calculations as these processes were considered too difficult to quantify (36). Another important uncertainty concerns the large variations in literature for devolatilization shock pressures for calcite and anhydrite, ranging between 20 GPa (77, 78) and >60 GPa (33). Artemieva et al. (36) used 60 GPa as the shock pressure for both porous (water saturated) and non-porous calcite when incipient decomposition starts. However, Bell et al. (33) showed with shock experiments that intact calcite is still found at these pressures. Hence, the total amount of CO_2_ released by the Chicxulub impact event over the entire crater must be considerably lower than expected from earlier calculations (425 ± 160 Gt CO_2_) (36). If the full range in CO_2_ back-reaction values of ∼30 to 80% is considered, derived from the isotopic study of natural impact samples of Martinez et al. (45) and based on a series of shock experiments on calcite by Agrinier et al. (32), while keeping the rest of the model parameters of Artemieva et al. (36) intact, the total amount of CO_2_ release is estimated to have been 230 ± 100 Gt CO_2_. Such new estimates of released climate active gases are crucial inputs for general circulation models to reconstruct land and sea surface temperatures (6, 39) and for quantifying ocean acidification (79) in the (hundreds) of kyrs post-Chicxulub impact. Therefore, updated estimates on the release of volatiles aid in unraveling the extinction and recovery dynamics of biotic groups across the K–Pg boundary and their link with carbon cycle perturbations.

## Materials and methods

### Sample materials

Thirty-six drill core samples are selected for this study, including bulk powders derived from previous Chicxulub projects (18, 21, 23, 35) and newly obtained powders based on careful microdrilling of specific carbonate clasts, areas of green carbonate matrix and a calcite vein. The sample set includes carbonate basement material, impact melt rocks, and suevites (polymict impact melt-bearing breccias), from four Yucatán drill cores on a transect from the northern Chicxulub peak ring toward the southern proximal ejecta blanket (Fig. 1A and B). The focus of this work lies on the IODP–ICDP Expedition 364 Hole M0077A drill core from the offshore peak ring (17) (Fig. 1). The petrography and geochemistry of the selected samples from the green marlstone, the “transitional unit,” suevite (polymict impact melt-bearing breccia) units and upper impact melt rock interval of this drill core are described in detail in Goderis et al. (80), Kaskes et al. (18), and de Graaff et al. (21). IODP–ICDP Exp. 364 samples from intervals below 737.5 mbsf are not included in this study due to a lack in carbonate components (21). In addition, we incorporated three suevite samples from the PEMEX Yucatán 6 drill core (Y6; N13 and N14) (23) and four samples from limestone intervals covering the entire depth range of the Cretaceous mega-block zone in the lower part of the ICDP Yaxcopoil-1 core (Yax-1), ranging in age from the Late Campanian to the Late Cenomanian (∼73–95 Ma) based on Sr-isotope stratigraphy (35). Lastly, two samples from the UNAM-7 drill core outside the outer and likely also exterior ring, at 126 km SE from the center of the Chicxulub structure (Fig. 1), are analyzed, which include a suevite and an underlying polymict impact breccia rich (50 in area %) in anhydrite clasts and poor (2.5 in area %) in silicate impact melt particles (56, 59).

### Petrography and element mapping

The identification and selection of Ca-bearing phases for isotopic analyses took place using a combined approach of detailed thin section petrography with high-resolution element mapping (*n* = 27; Figs. S1–S7). Thin sections (30 µm thick) were examined using a Zeiss (Carl Zeiss GmbH, Jena, Germany) Axioscope 5 TL/RL polarizing microscope equipped with an Axiocam 208 camera, at Vrije Universiteit Brussel, Belgium (VUB). The nature of carbonate phases in a selection of thin sections (*n* = 8) was also investigated using a JEOL JSM-IT300 (JEOL Ltd., Tokyo, Japan) scanning electron microscope equipped with an energy dispersive X-ray spectrometer (SEM-EDS) at VUB, applying an acceleration voltage of 15.0 kV, a resolution of 768 × 1,024 pixels, and a pixel dwell time of 2,000–5,000 µs for EDS element mapping (following Kaskes et al. 18,; Figs. 4, and S2–S5). In addition, both thin sections and polished thick sections were studied by non-destructive micro-X-ray fluorescence (µXRF) mapping at VUB using an M4 Tornado benchtop µXRF surface scanner (Bruker nano GmbH, Berlin, Germany) equipped with a Rh tube as X-ray source and two XFlash 430 Silicon Drift detectors. All measurements were carried out under near vacuum conditions (20 mbar) with a spot size of 25 μm, a spatial resolution of 25–30 μm and an integration time of 1 ms per pixel (following Kaskes et al. [18, 81]). The obtained qualitative multi-element maps and semi-quantitative single-element µXRF heatmaps (Fig. S1) were used to select the carbonate phases for micro-sampling. CaCO_3_ content (in volatile-free, normalized wt%, see Figs. 3C and S12, and Table S1) of regions of interests on the µXRF maps was determined using polygonization in the M4 software and by applying a Standardless Fundamental Parameters quantification as outlined in Kaskes et al. (81).

### Conventional and clumped stable isotope analysis

The stable carbon (δ^13^C), oxygen (δ^18^O), and clumped (Δ_47_) isotope analysis of carbonate powders (varying in weight from 0.5 to 3.5 mg depending on the carbonate content) are measured in the Archaeology, Environmental Changes and Geo-Chemistry (AMGC) clumped laboratory of the VUB, using a Nu Instruments Perspective-IS stable isotope ratio mass spectrometer (SIRMS) in conjunction with a Nu-Carb carbonate sample preparation system, as described in detail in De Vleeschouwer et al. (11). We performed isotope measurements on various carbonate phases (Figs. S1–S7), comprising green matrix material (green schlieren and smooth green textures) in brecciated impact melt rocks, calcite veins, and (fossiliferous) carbonate clasts in suevites, in addition to bulk powders. The δ^13^C data are expressed relative to VPDB and δ^18^O results expressed relative to both VPDB and VSMOW. Analyses and results are monitored in the laboratory using the Easotope software (82). The carbonate standard ETH-2 is systematically measured and compared to InterCarb values (62) to ensure the quality control of our measurements. The raw measured Δ_47_ values were processed using the IUPAC (International Union of Pure and Applied Chemistry) Brand's isotopic parameters (83) and converted to the I-CDES 90°C scale, using the most recent values for the ETH-1, ETH-3, and ETH-4 carbonate reference materials (62) within the ClumpyCrunch software (83, 84). The average Δ_47_ values for each sample are converted into temperatures using the Anderson et al. (85) calibration. Both analytical and calibration uncertainties are propagated to calculate the final uncertainties on temperatures. The full sample list with δ^18^O, δ^13^C, Δ_47_, and *T*(Δ_47_) data can be found in Table S1. In addition, conventional and clumped-isotope data from Bralower et al. (30) (*n* = 19), which focused on the top part of the impactite sequence of the IODP–ICDP Exp. 364 drill core (uppermost suevite, transitional unit, green marlstone, and Paleogene sediments), are used in this study for comparison and show good overlap with three control samples of these lithological units measured in this study (Figs. 2A, 3, S8A, and S9A). Using the ETH 1–4 reported for their dataset, the sample data were reprocessed following the InterCarb procedure (62), and the recalculated Δ_47_ values were converted into temperatures using the same calibration of Anderson et al. (85). The recalculated *T*(Δ_47_) values were on average 4–5°C warmer for the Paleogene limestones and the transitional unit, and on average ∼7°C colder for the upper suevite interval compared to the original values reported by Bralower et al. (30). The Δ_47_ values from (14) could not be recalculated as no ETH standards were measured in that study.

## Supplementary Material

pgad414_Supplementary_Data

## Data Availability

All data are included in the manuscript and/or Supplementary material. Core samples from the IODP–ICDP Expedition 364 M0077 drill core are available upon request from the International Ocean Discovery Program.
