# Supplementary material for: Hot carbonates deep within the Chicxulub impact structure
Source: PNAS Nexus. 2024 Jan 11;3(1):pgad414. doi: 10.1093/pnasnexus/pgad414 (PMC10783646; doi:10.1093/pnasnexus/pgad414)
Supplement: pgad414_Supplementary_Data [file pgad414_supplementary_data.pdf]

PNAS nexus

Supplementary Information for
Hot carbonates deep within the Chicxulub impact structure

Pim Kaskes[1,2*], Marta Marchegiano[1,3], Marion Peral[1,4], Steven Goderis[1], and Philippe Claeys[1]

[1] Research Unit: Archaeology, Environmental changes & Geo-Chemistry (AMGC), Vrije Universiteit Brussel, Pleinlaan 2, 1050 Brussels, Belgium.

[2] Laboratoire G-Time, Université Libre de Bruxelles, Av. F.D. Roosevelt 50, 1050 Brussels, Belgium.

[3] Department of Stratigraphy and Paleontology, University of Granada, 18071 Granada, Spain.

[4] CNRS, Bordeaux INP, EPOC, UMR 5805, Université de Bordeaux, F-33600 Pessac, France

*Pim Kaskes

Email: pim.kaskes@vub.be

This PDF file includes:

Figures S1 to S12
Table S1

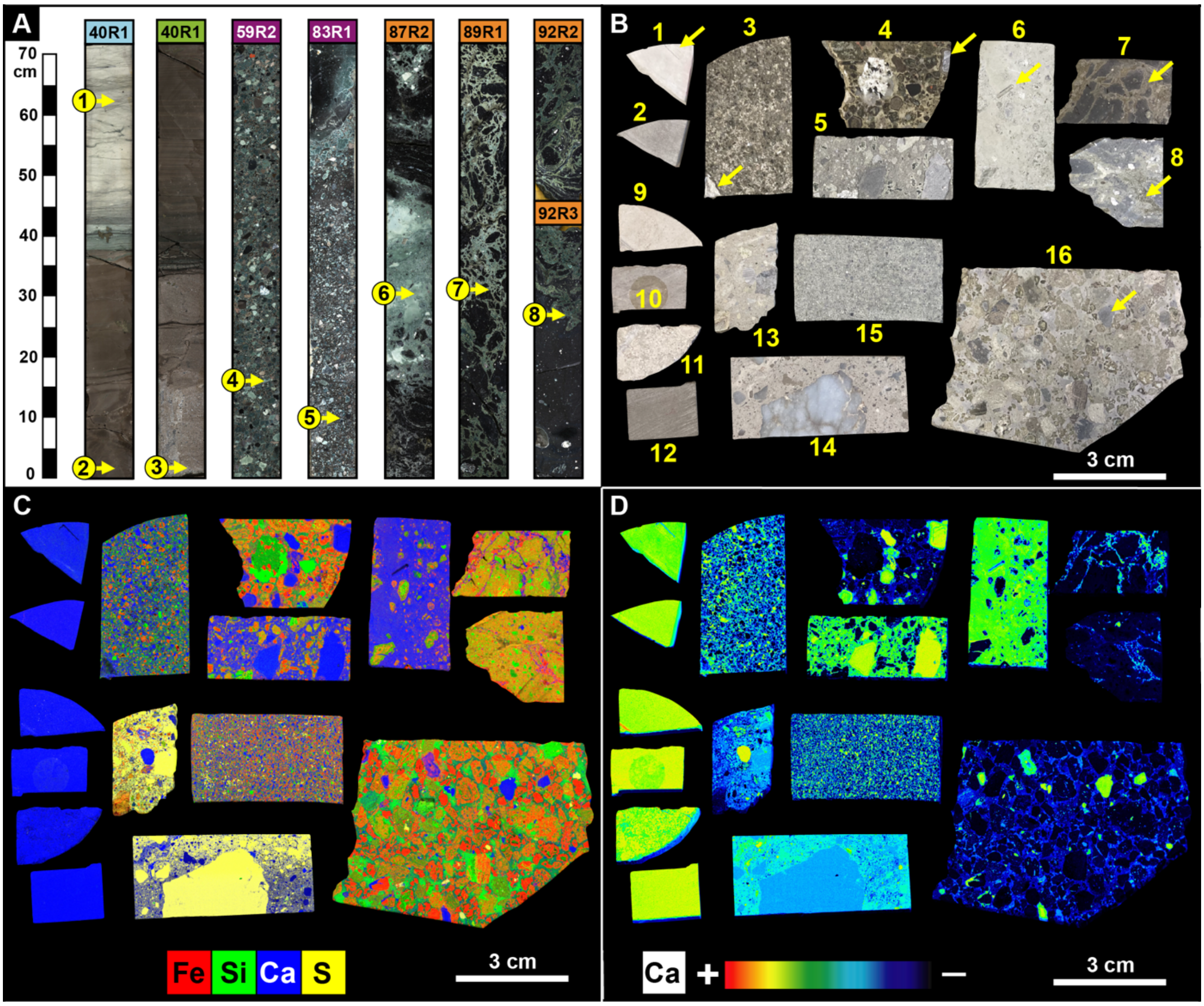

Fig. S1. Representative samples of impact and target lithologies from Chicxulub drill cores. These samples are examined by high-resolution micro-X-ray fluorescence (µXRF) mapping to select carbonate phases for this isotopic study. See 'Materials and Methods' for more information on these samples. **(A)** Overview of selected halfcore photographs of the IODP-ICDP Expedition 364 drill core, which covers important parts of the sample sets. Sample location of hand specimens in B are indicated and colors of the core labels match the lithology legend in Fig. 2. **(B)** Macrophotograph of a subselection of the sample set, yellow arrows indicate specific locations of carbonate phases that were microdrilled, on top of the bulk powder that was obtained for each sample. **(C)** Multi-element µXRF map showing the distributions of Fe, Si, Ca, and S in the various core samples. **(D)** Single-element µXRF heatmap showing the relative distribution of Ca. The numbers of the drill core samples, derived from IODP-ICDP Expedition 364, ICDP Yaxcopoil-1, UNAM-7, and PEMEX Yucatán 6, are explained below. In Table S1, a full list of the studied samples can be found. 1. Post-impact limestone (IODP-ICDP Exp. 364; 616.29 mbsf). 2. Transitional unit - siltstone (IODP-ICDP Exp. 364; 617.04 mbsf). 3. Upper (layered) suevite (IODP-ICDP Exp. 364; 617.67 mbsf). 4. Middle (graded) suevite (IODP-ICDP Exp. 364; 675.91 mbsf). 5. Basal (non-graded) suevite (IODP-ICDP Exp. 364; 713.23 mbsf). 6. Brecciated impact melt rock (IODP-ICDP Exp. 364; 721.45 mbsf). 7. Brecciated impact melt rock (IODP-ICDP Exp. 364; 726.21 mbsf). 8. Brecciated impact melt rock (IODP-ICDP Exp. 364; 737.51 mbsf). 9. Pre-impact limestone basement (ICDP Yax-1; 914.07 mbs). 10. Pre-impact limestone basement (ICDP Yax-1; 1002.44 mbs). 11. Pre-impact limestone basement (ICDP Yax-1; 1182.38 mbs). 12. Pre-impact limestone basement (ICDP Yax-1; 1476.64 mbs). 13. Suevite, proximal ejecta blanket (UNAM-7; 267.45 mbs). 14. Melt-poor polymict lithic impact breccia (UNAM-7; 381.40 mbs). 15. Upper suevite (PEMEX Y6; 1103 mbs). 16. Middle suevite (PEMEX Y6; 1208 mbs).

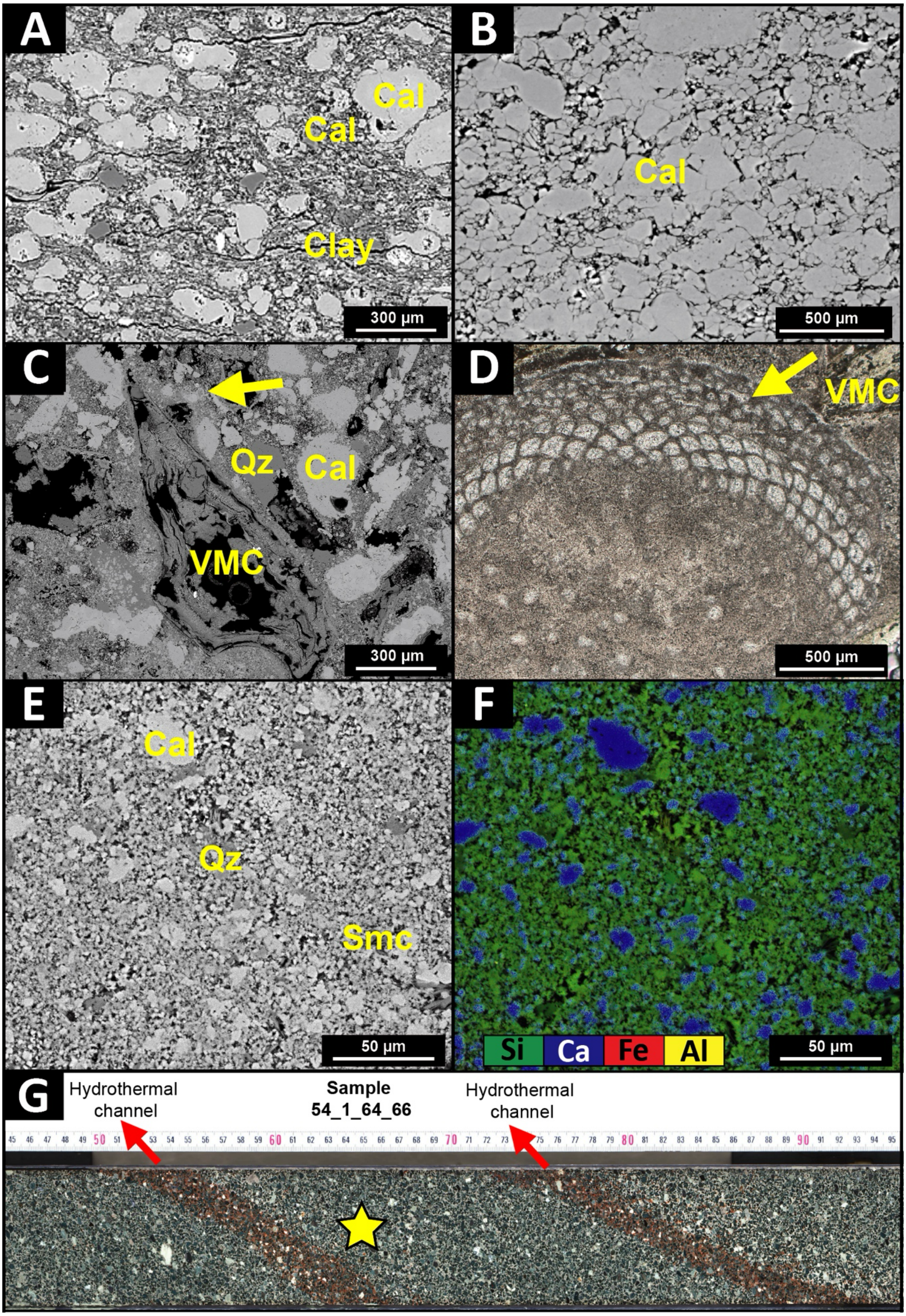

Fig. S2. Lithological and petrographical characteristics of representative carbonate phases within the upper part of the IODP-ICDP Expedition 364 impactite sequence. (A) SEM backscatter image of rounded foraminiferal calcite (Cal) grains, quartz (Qz) and phyllosilicates (Clay) in the upper part of the green marlstone (616.56 mbsf; modified from: (*30*)). **(B)** SEM backscatter image of the

transitional unit with a matrix of (sub)angular grains of micrite, interpreted to have formed from decarbonation-carbonation processes (616.94 mbsf; modified from: (*30*)). **(C)** SEM backscatter image of the upper (layered) suevite with abundant vitric silicate melt particles (VMC) embedded in a clastic matrix dominated by subrounded carbonate (~40 area %) and quartz grains (617.67 mbsf). **(D)** Plane polarized light (PPL) microphotograph of the middle (graded) suevite with a fossiliferous carbonate clast containing the large benthic foraminifera *Omphalocyclus* sp. (640.86 mbsf; modified from: (*18*)). Foraminifera are indicated with yellow arrows. **(E-F)** Middle (graded) suevite with clastic matrix containing subrounded carbonate clasts (~25 area %), quartz and feldspar (~25%) and phyllosilicaties, mostly smectite (Smc) (~50%), visualized by a SEM-EDS overview showing the distribution of Si, Ca, Fe and Al phases (675.91 mbsf; modified from: (*18*)). **(G)** Overview of core section 54R1 showing two red-orange-brown zones that have been interpreted as hydrothermal channels (*28*). Graded suevite sample 54_1_64_66 is located in very close proximity to these hydrothermal channels, which could explain the high $T(\Delta_{47})$ value of 135.6°C ± 21°C (1 SE).

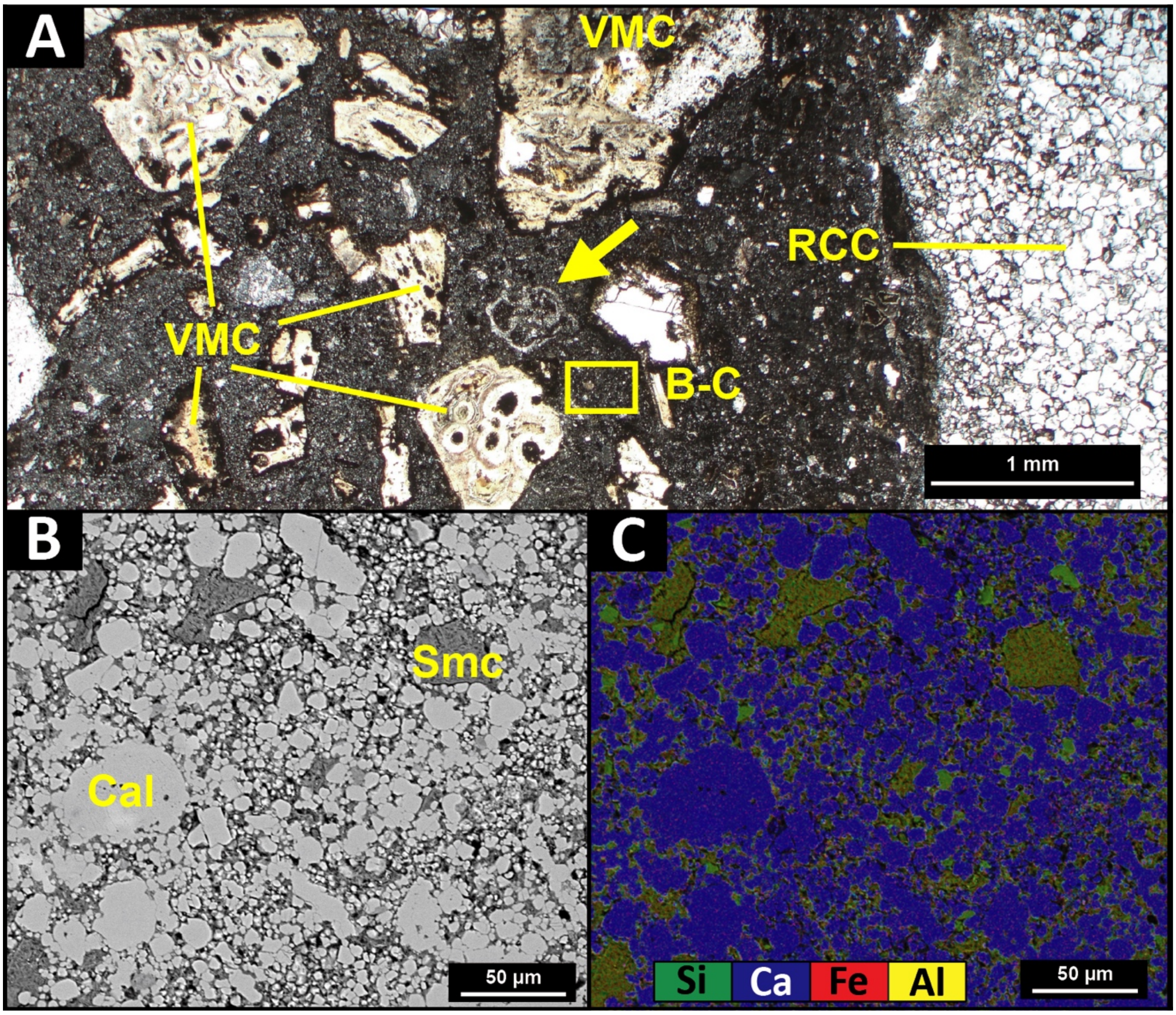

Fig. S3. Petrography of representative carbonate phases within the non-graded suevite interval of the IODP-ICDP Expedition 364 drill core. (A) Lower (non-graded) suevite with abundant vitric silicate melt particles (VMC), a large, recrystallized carbonate clast (RCC) and partly recrystallized but still recognizable planktic foraminifera (see yellow arrow) in the matrix (715.09 mbsf; modified from: (*19*)). (**B-C)** SEM-EDS overview of the same sample (715.09 mbsf) showing a clear Ca rich matrix with subrounded calcite (Cal) particles and some smectite (Smc).

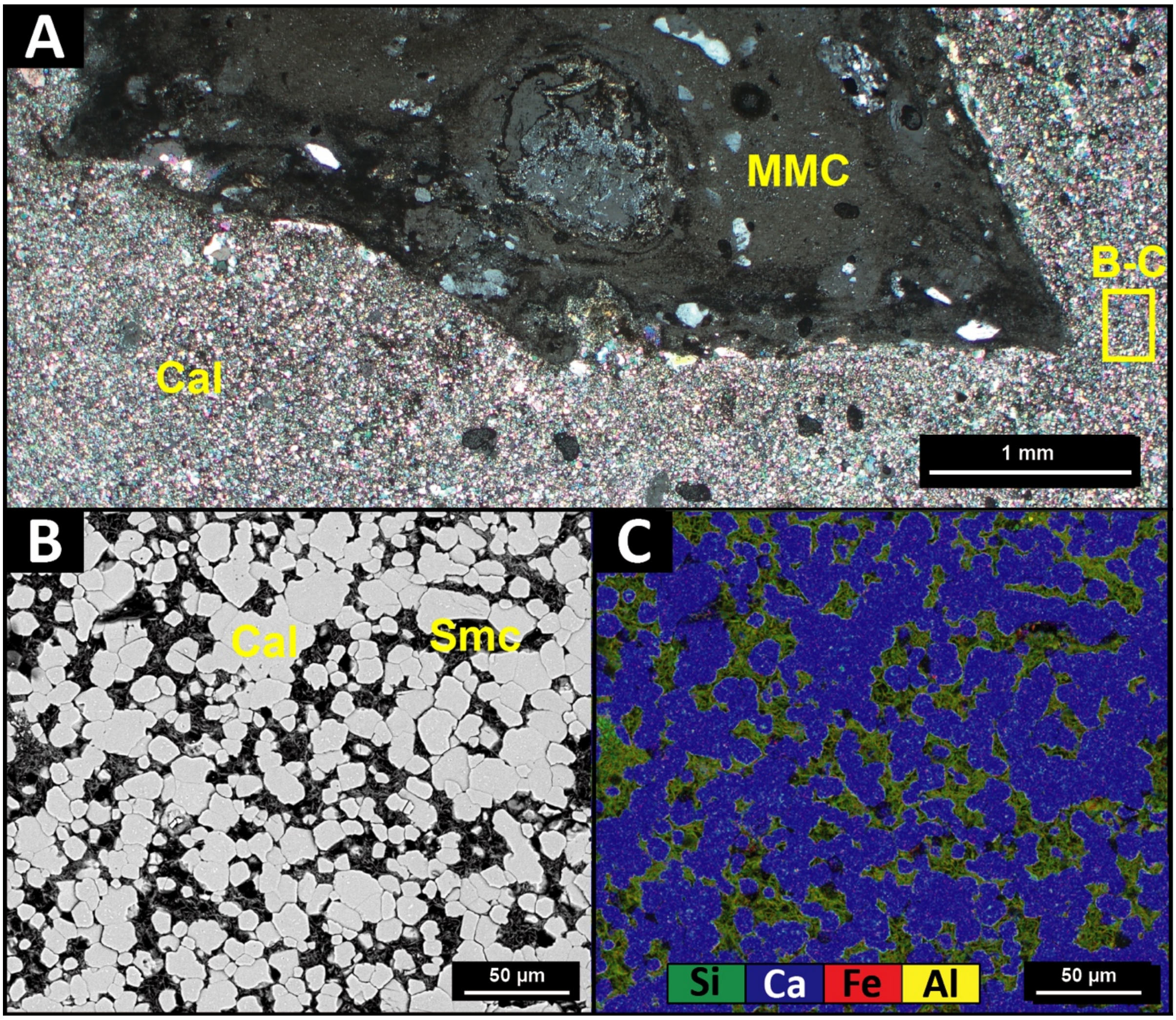

Fig. S4. Petrography of representative carbonate phases within impact melt rock interval 721.45 mbsf of the IODP-ICDP Expedition 364 drill core. (A) Cross polarized light (XPL) microphotograph image of a brecciated impact melt rock with a large microcrystalline melt clast (MMC) floating in a calcite matrix (721.45 mbsf). **(B-C)** SEM-EDS overview of the same brecciated impact melt rock sample displaying clear equigranular calcite, this sample is associated with the highest T(Δ_{47}) value (327°C ± 33°C (1 SE)).

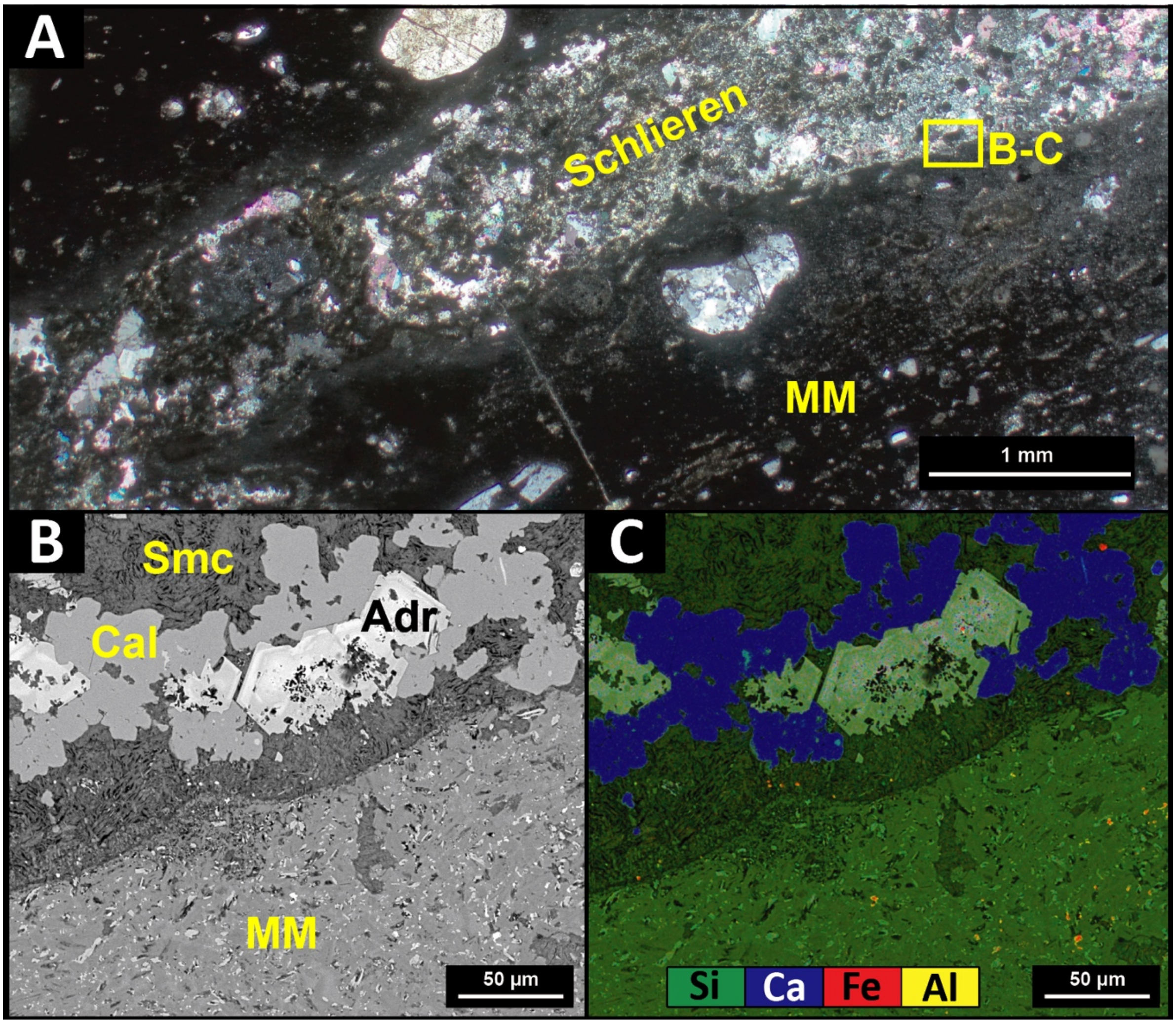

Fig. S5. Petrography of representative carbonate phases within impact melt rock interval 726.21 mbsf of the IODP-ICDP Expedition 364 drill core. (A) Cross polarized light (XPL) microphotograph image of the contact between a green schlieren zone and microcrystalline impact melt rock (MM) (726.21 mbsf). **(B-C)** SEM-EDS overview of the same sample showing that the cracks within the green schlieren zone are filled with calcite, grossular-rimmed andradite-garnet (Adr) and smectite-group clays (most likely dominated by saponite).

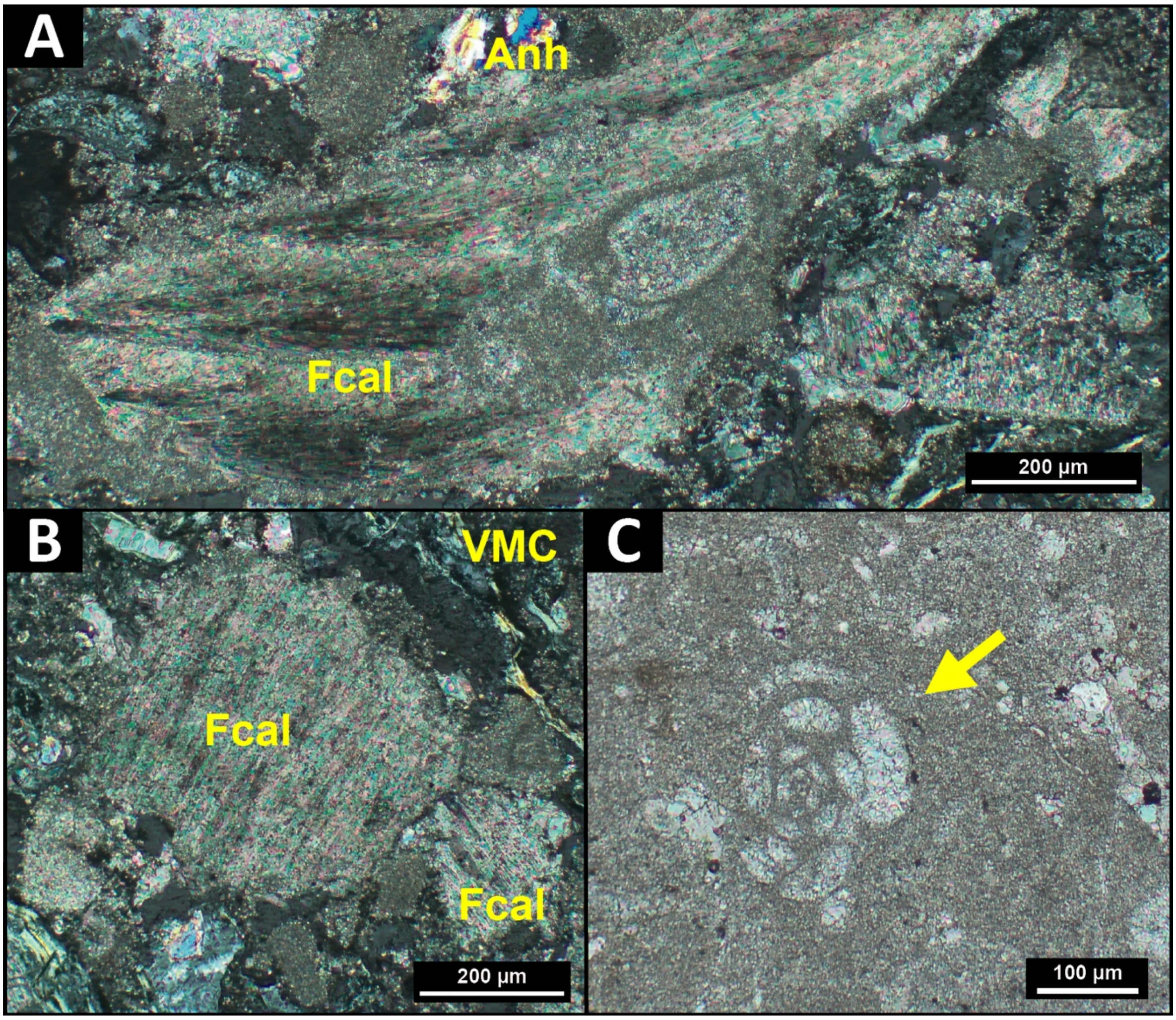

Fig. S6. Petrography of representative carbonate phases within the PEMEX Yucatán-6 (Y6) and ICDP Yacxopoil-1 (Yax-1) drill cores. (A-B) XPL microphotograph of feathery calcite (Fcal) in the upper suevite of Y6 (N13; 1103 mbs), together with anhydrite (Anh) and vitric melt clasts (VMC). **(C)** PPL microphotograph of Yax-1 pre-impact limestone basement (907.49 mbs) with poorly preserved foraminifera (see yellow arrow).

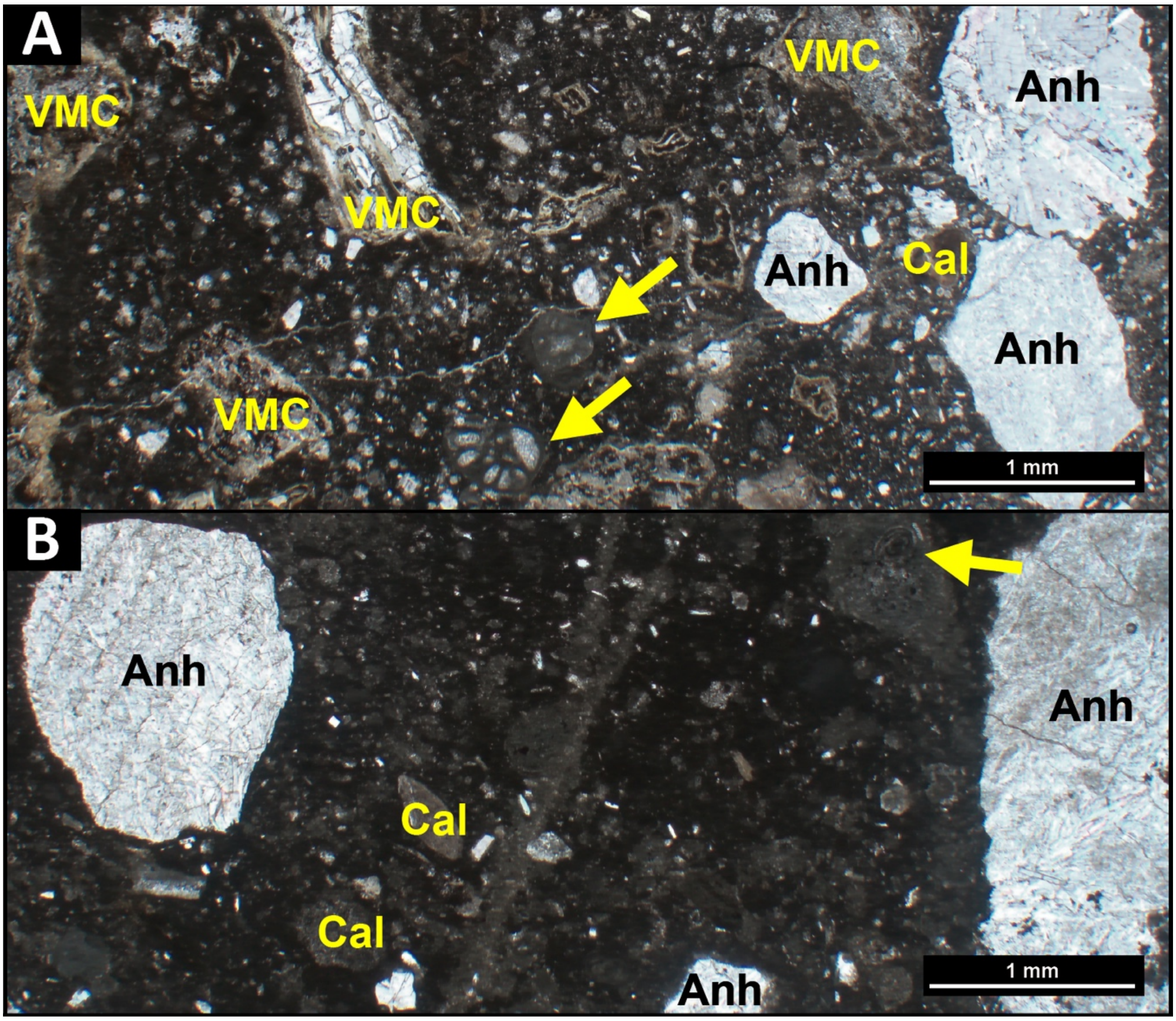

Fig. S7. Petrography of representative carbonate phases within the UNAM-7 drill core outside of the Chicxulub impact crater. (A) PPL microphotograph of the UNAM-7 suevite with clasts of vitric silicate melt (VMC), anhydrite (Anh), and carbonate (Cal) (267.45 mbsf). **(B)** PPL microphotograph of the underlying UNAM-7 polymict lithic breccia with clasts of anhydrite and carbonate (381.45 mbsf). Foraminifera are indicated with yellow arrows.

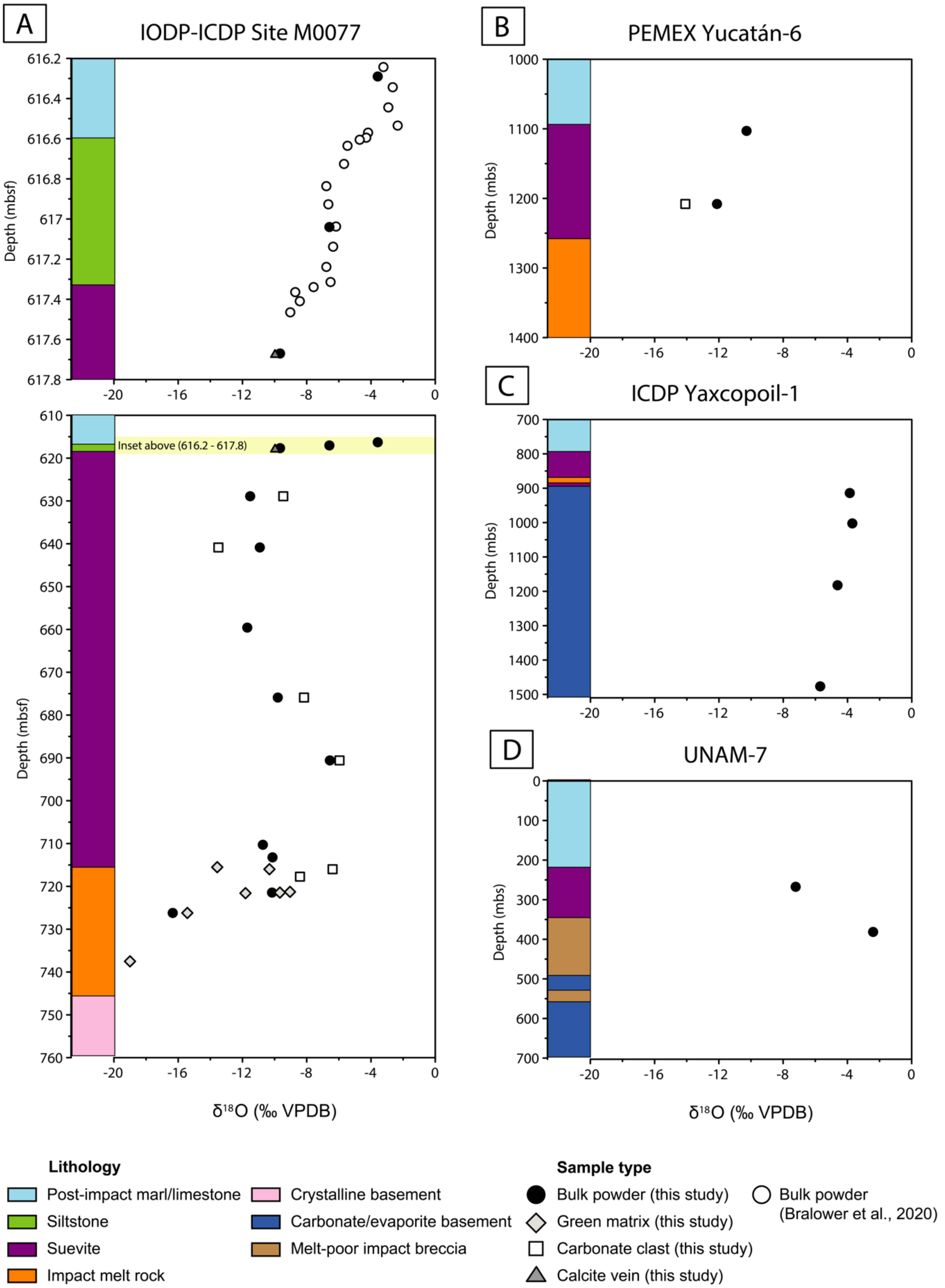

Fig. S8. Stratigraphic overview showing δ^{18}O data versus depth, lithology, and sample type of the four Chicxulub drill cores. (A) IODP-ICDP Exp. 364 drill core from Site M0077, with in the inset between 616.2 – 617.8 meters below seafloor (mbsf) also incorporating δ^{18}O data from (30); **(B)** PEMEX Yucatán-6 (Y6); **(C)** ICDP Yaxcopoil-1 (Yax-1); **(D)** UNAM-7. VPDB – Vienna Peedee belemnite.

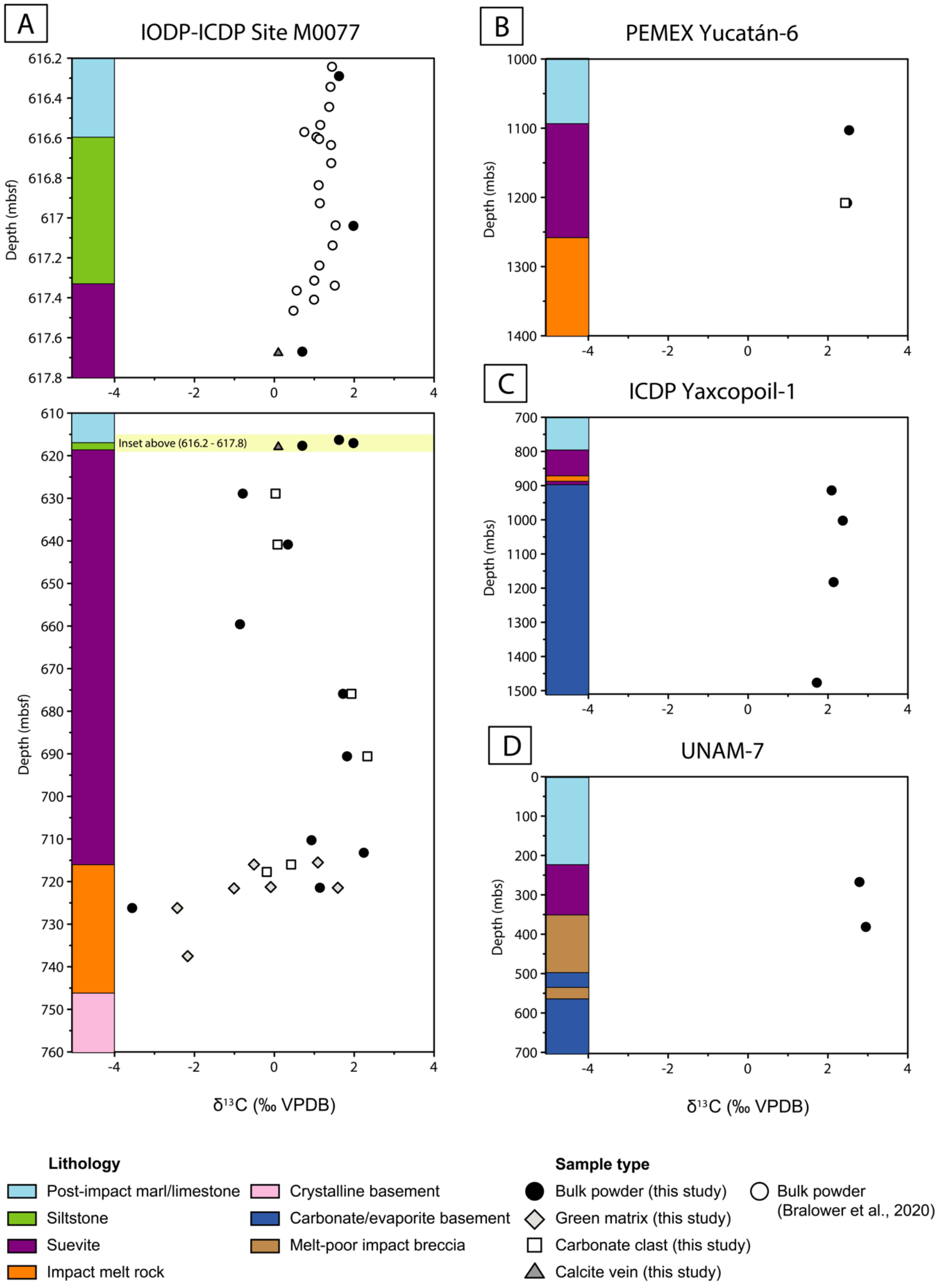

Fig. S9. Stratigraphic overview showing $\delta^{13}C$ data versus depth, lithology, and sample type of the four Chicxulub drill cores. (A) IODP-ICDP Exp. 364 drill core from Site M0077, with in the inset between 616.2 – 617.8 meters below seafloor (mbsf) also incorporating $\delta^{13}C$ data from (30); **(B)** PEMEX Yucatán-6 (Y6); **(C)** ICDP Yaxcopoil-1 (Yax-1); **(D)** UNAM-7. VPDB – Vienna Peedee belemnite.

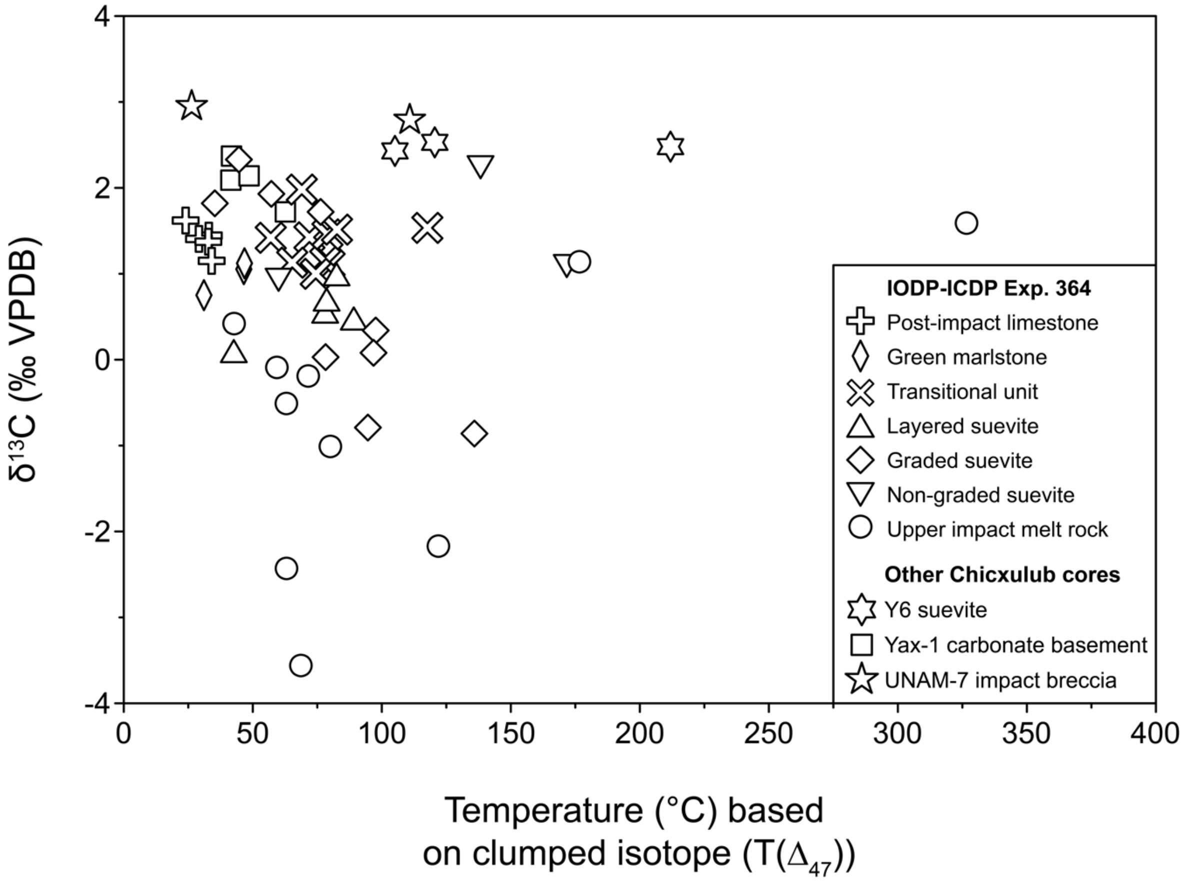

Fig. S10. Relationship between $\delta^{13}C$ data and $T(\Delta_{47})$. No correlation has been found between the two datasets. VPDB – Vienna Peedee belemnite.

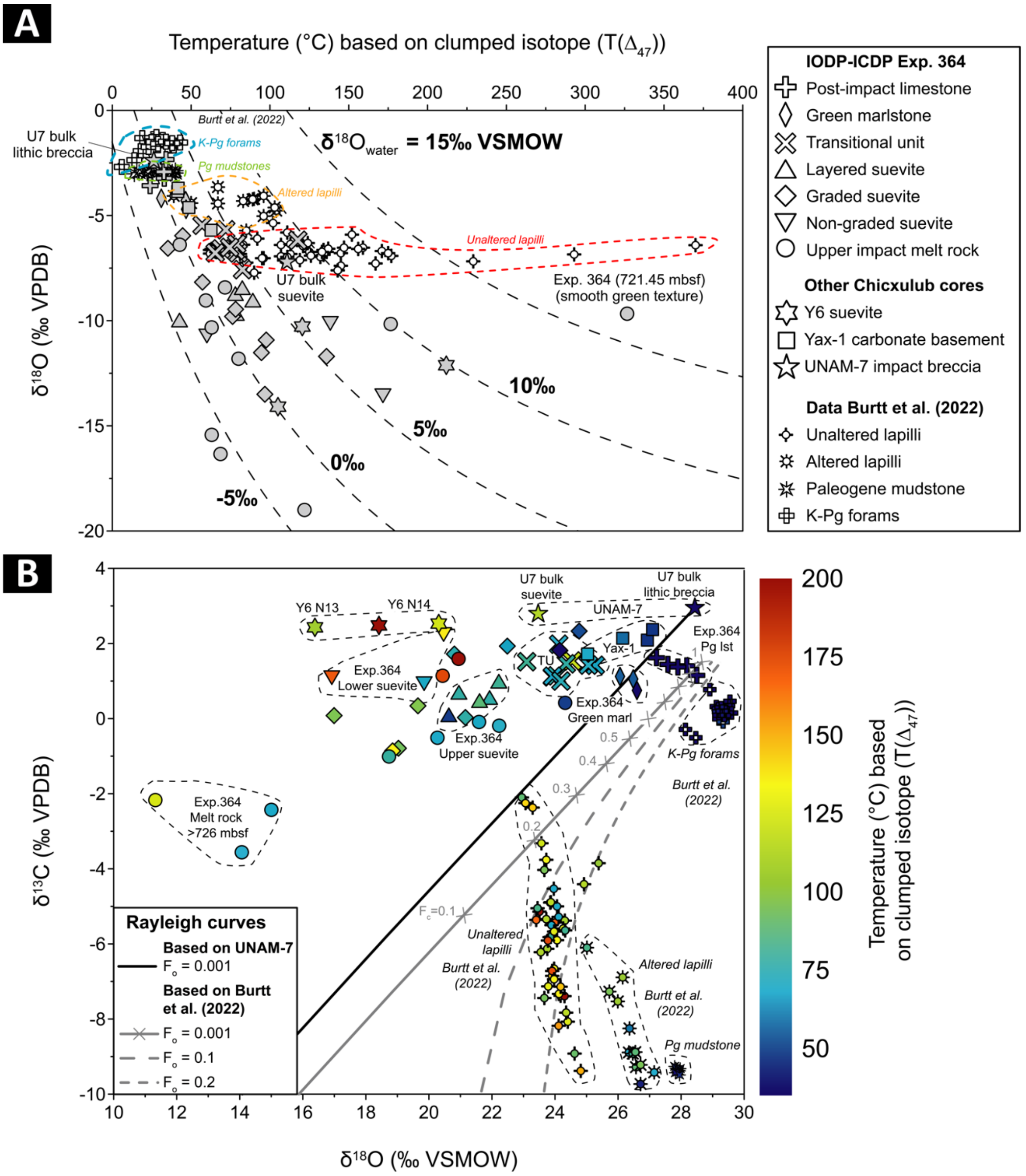

Fig. S11. Isotopic cross relationships of Chicxulub core and impact ejecta material. (A) Relationship between $\delta^{18}O$ data and average $T(\Delta_{47})$ data with the symbols referring to the lithological units from the four different drill cores (in grey), including the dataset of Burtt et al. (14) (with white symbols and highlighted with colored fields). Dashed lines are constant $\delta^{18}O_{water}$ (VSMOW) values of possible diagenetic fluids based on the carbonate-water equilibrium relationships of (*41*). Note that Burtt et al. (*14*) used a different standardization and the $T(\Delta_{47})$ comparison here should be seen as relative, as it is not possible to recalculate the absolute temperatures of Burtt et al. (*14*) using the universally accepted I-CDES reference frame (*62*). **(B)** Isotopic cross plot showing $\delta^{13}C$ (VPDB) versus $\delta^{18}O$ (VSMOW) data with colors indicating the $T(\Delta_{47})$ data. Four Rayleigh trends are highlighted in this plot that represent fractionation during impact decarbonation as expressed by varying mole fractions of oxygen (F_o) (following (*14, 42*). The extent of the reaction (F_c) is plotted along one Rayleigh curve to show the extent of decarbonation. In addition, the K-Pg ejecta dataset from (*14*) is shown, in which the accretionary lapilli data largely plots within fields of F_o of ~0.1-0.2. This indicates that the formation of the lapilli was steered by decarbonation, but that the decarbonation process was not fully complete (*14*). The data from the current study plots largely above the Rayleigh curves, suggesting that decarbonation following atmospheric processes is not a major factor in explaining the isotopic trends and therefore,

other processes, such as mixing with silicate components and hydrothermal alteration, are more dominant in the Chicxulub proximal impactite deposition. VPDB – Vienna Peedee belemnite. VSMOW – Vienna Standard Mean Ocean Water.

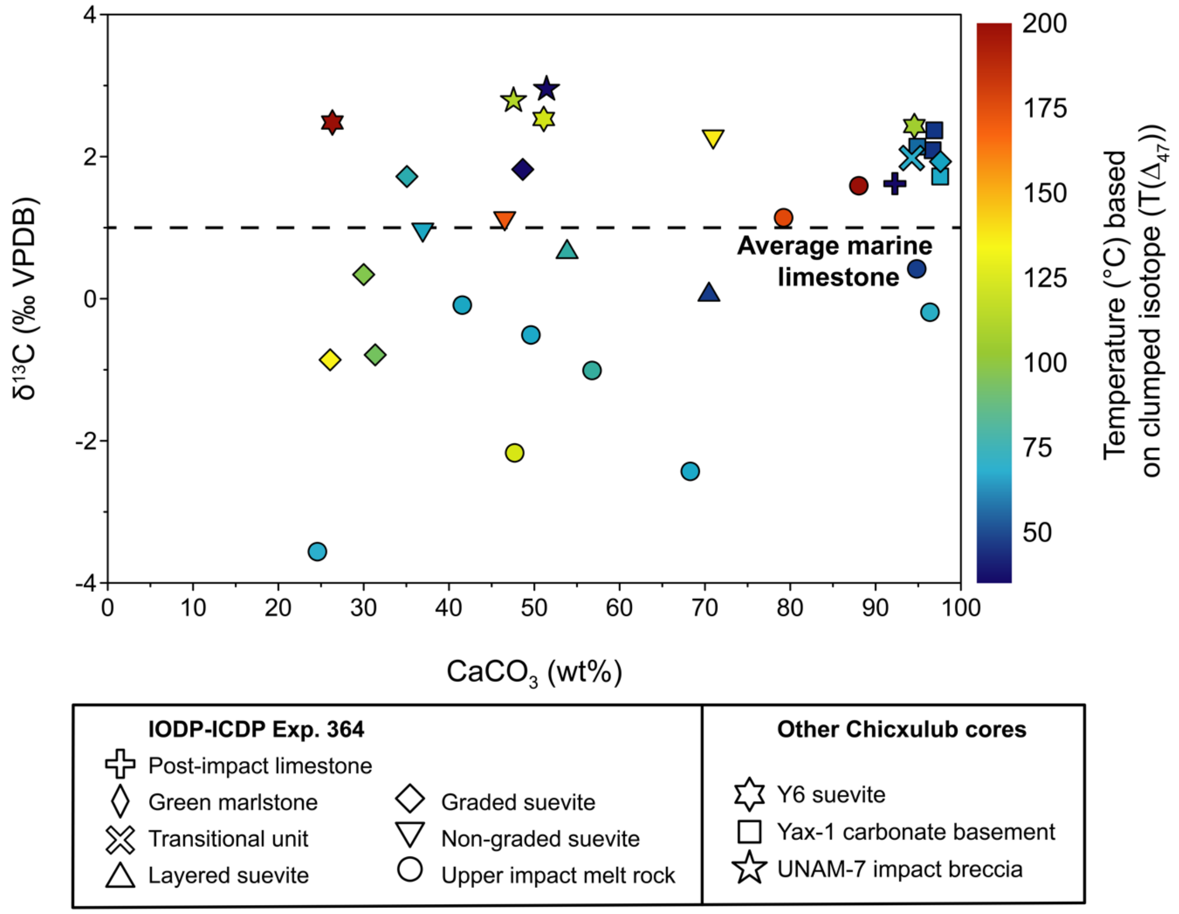

Fig. S12. Relationship between $\delta^{13}C$ and $CaCO_3$ content. Colors indicate the T(Δ_{47}) data. The dashed line is representing the average $\delta^{13}C$ signal for marine carbonate (~1 ‰ VPDB; (*44*)). Most of the high T(Δ_{47}) samples plot above this value, suggesting that thermal impact processes are responsible for the more positive $\delta^{13}C$ compositions in these crater lithologies.

Table S1. Overview of the analyzed Chicxulub samples and their stable oxygen, carbon, and clumped isotope data, together with their calcium carbonate content.

Drill core	Core sample ID	Depth (mbs or mbsf)	Core and lithological unit	Lithology	$CaCO_3$ (wt% based on µXRF)	Sample type for isotope analysis	Lab ID	n° replicates	$\delta^{18}O$ (‰ VPDB)	$\delta^{18}O$ (‰ VSMOW)	$\delta^{13}C$ (‰VDPB)	Δ_{47}	SE	T (°C)	SE+/-	2SE +/-
ICDP Yaxcopoil-1	YAX_1505-2B	914.07	Yax-1 carbonate target rock	Carbonate target rock	96.69	Bulk powder	PIM3-7	3	-3.86	26.93	2.09	0.5489	0.0178	41.5	5.4	10.7
ICDP Yaxcopoil-1	YAX_1545	1002.44	Yax-1 carbonate target rock	Carbonate target rock	96.88	Bulk powder	PIM3-8	3	-3.70	27.10	2.37	0.5483	0.0178	41.8	5.4	10.7
ICDP Yaxcopoil-1	YAX_1612A	1182.38	Yax-1 carbonate target rock	Carbonate target rock	94.89	Bulk powder	PIM3-9	3	-4.62	26.15	2.14	0.5318	0.0181	48.6	5.8	11.7
ICDP Yaxcopoil-1	Yax_1716a	1476.64	Yax-1 carbonate target rock	Carbonate target rock	97.57	Bulk powder	PIM3-10	3	-5.70	25.03	1.72	0.5008	0.0179	62.6	6.5	13.1
PEMEX-Yucatán-6	Y6_N13_P9	1103.00	Y6 suevite	Suevite	51.10	Bulk powder	PIM3-11	3	-10.28	20.31	2.53	0.4063	0.0177	120.5	10.3	20.5
PEMEX-Yucatán-6	Y6_N14_11A	1208.00	Y6 suevite	Suevite	26.33	Bulk powder	PIM3-13	3	-12.12	18.42	2.48	0.3202	0.0175	211.9	18.6	37.1
PEMEX-Yucatán-6	Y6_N14_11A	1208.00	Y6 suevite	Suevite	94.54	Carbonate clast	PIM3-14	3	-14.09	16.38	2.43	0.4273	0.0177	105.1	9.1	18.3
UNAM-7	UNAM7_381.40-381.50_D	381.45	UNAM-7 impact breccia	Melt-poor impact breccia	51.43	Bulk powder	PIM3-15	3	-2.40	28.44	2.95	0.5900	0.0180	26.3	4.7	9.4
UNAM-7	UNAM7_267.40-267.50_C	267.45	UNAM-7 impact breccia	Suevite	47.56	Bulk powder	PIM3-16	3	-7.22	23.47	2.79	0.4192	0.0178	110.8	9.6	19.2
IODP-ICDP Exp. 364	40_1_10_11	616.29	IODP-ICDP Exp. 364 post-impact limestone	Chalk	92.26	Bulk powder	PIM2-1	3	-3.58	27.22	1.62	0.5969	0.0180	24.0	4.6	9.2
IODP-ICDP Exp. 364	364 77A 40R-1 0-1 cm	616.24	IODP-ICDP Exp. 364 post-impact limestone	Chalk		Bulk powder (Bralower et al., 2020)		1	-3.23	27.58	1.44	0.5713	0.0284	33.0	8.6	17.1
IODP-ICDP Exp. 364	364-77A-40R-1 10-11cm	616.34	IODP-ICDP Exp. 364 post-impact limestone	Chalk		Bulk powder (Bralower et al., 2020)		2	-2.65	28.18	1.41	0.5819	0.0208	29.1	5.8	11.5
IODP-ICDP Exp. 364	364 77A 40R-1 20-21 cm	616.44	IODP-ICDP Exp. 364 post-impact limestone	Chalk		Bulk powder (Bralower et al., 2020)		2	-2.92	27.90	1.37	0.5718	0.0208	32.8	6.0	11.9

IODP-ICDP Exp. 364	364-77A 40R-1 29-30 cm	616.53	IODP-ICDP Exp. 364 post-impact limestone	Chalk		Bulk powder (Bralower et al., 2020)		2	-2.34	28.50	1.15	0.5683	0.0207	34.1	6.0	12.0
IODP-ICDP Exp. 364	364-77A 40R-1 32-34 cm	616.57	IODP-ICDP Exp. 364 Green marlstone	Marl		Bulk powder (Bralower et al., 2020)		2	-4.18	26.60	0.75	0.5765	0.0207	31.1	5.8	11.7
IODP-ICDP Exp. 364	364-77A 40R-1 35-36 cm	616.59	IODP-ICDP Exp. 364 Green marlstone	Marl		Bulk powder (Bralower et al., 2020)		2	-4.30	26.48	1.05	0.5366	0.0205	46.5	6.7	13.4
IODP-ICDP Exp. 364	364-77A 40R-1 36-37 cm	616.61	IODP-ICDP Exp. 364 Green marlstone	Marl		Bulk powder (Bralower et al., 2020)		2	-4.70	26.06	1.12	0.5360	0.0139	46.8	4.1	8.1
IODP-ICDP Exp. 364	364-77A 40R-1 39-40 cm	616.64	IODP-ICDP Exp. 364 Transitional unit	Siltstone		Bulk powder (Bralower et al., 2020)		1	-5.46	25.28	1.42	0.5128	0.0170	57.0	5.8	11.6
IODP-ICDP Exp. 364	364-77A 40R-1 48-49 cm	616.73	IODP-ICDP Exp. 364 Transitional unit	Siltstone		Bulk powder (Bralower et al., 2020)		2	-5.68	25.06	1.42	0.4823	0.0202	72.0	8.2	16.4
IODP-ICDP Exp. 364	364-77A 40R-1 59-60 cm	616.84	IODP-ICDP Exp. 364 Transitional unit	Siltstone		Bulk powder (Bralower et al., 2020)		2	-6.78	23.92	1.11	0.4679	0.0202	79.8	8.8	17.5
IODP-ICDP Exp. 364	364-77A-40R-1 68-69 cm	616.93	IODP-ICDP Exp. 364 Transitional unit	Siltstone		Bulk powder (Bralower et al., 2020)		1	-6.65	24.05	1.14	0.4710	0.0280	78.1	12.6	25.2
IODP-ICDP Exp. 364	364-77A-40R-1 79-80 cm	617.04	IODP-ICDP Exp. 364 Transitional unit	Siltstone		Bulk powder (Bralower et al., 2020)		1	-6.18	24.54	1.53	0.4100	0.0280	117.7	17.1	34.2
IODP-ICDP Exp. 364	40_1_80_81	617.04	IODP-ICDP Exp. 364 Transitional unit	Siltstone	94.24	Bulk powder	PIM2-2	3	-6.59	24.12	1.98	0.4880	0.0175	69.0	6.7	13.4
IODP-ICDP Exp. 364	364 77A 40R-1 89-90 cm	617.14	IODP-ICDP Exp. 364 Transitional unit	Siltstone		Bulk powder (Bralower et al., 2020)		2	-6.36	24.36	1.46	0.4694	0.0202	78.9	8.7	17.4
IODP-ICDP Exp. 364	364 77A 40R-1 99-100 cm	617.24	IODP-ICDP Exp. 364 Transitional unit	Siltstone		Bulk powder (Bralower et al., 2020)		2	-6.78	23.92	1.13	0.4954	0.0202	65.3	7.8	15.5
IODP-ICDP Exp. 364	364-77A 40R-1 106.5-107.5 cm	617.31	IODP-ICDP Exp. 364 Transitional unit	Siltstone		Bulk powder (Bralower et al., 2020)		2	-6.52	24.19	1.00	0.4777	0.0202	74.4	8.4	16.8

IODP-ICDP Exp. 364	364-77A 40R-1 109-110 cm	617.34	IODP-ICDP Exp. 364 Transitional unit	Siltstone		Bulk powder (Bralower et al., 2020)		2	-7.57	23.10	1.51	0.4629	0.0202	82.6	9.0	18.0
IODP-ICDP Exp. 364	364-77A 40R-1 111-113 cm	617.36	IODP-ICDP Exp. 364 Bedded suevite unit	Suevite		Bulk powder (Bralower et al., 2020)		2	-8.71	21.93	0.56	0.4710	0.0201	78.1	8.6	17.2
IODP-ICDP Exp. 364	364-77A 40R-1 116-117 cm	617.41	IODP-ICDP Exp. 364 Bedded suevite unit	Suevite		Bulk powder (Bralower et al., 2020)		2	-8.43	22.22	0.99	0.4632	0.0201	82.5	8.9	17.8
IODP-ICDP Exp. 364	364-77A 40R-1 121-123 cm	617.46	IODP-ICDP Exp. 364 Bedded suevite unit	Suevite		Bulk powder (Bralower et al., 2020)		2	-9.02	21.61	0.48	0.4519	0.0201	89.1	9.4	18.8
IODP-ICDP Exp. 364	40_2_0_3	617.67	IODP-ICDP Exp. 364 Bedded suevite unit	Suevite	53.84	Bulk powder	PIM2-3	3	-9.66	20.95	0.70	0.4700	0.0174	78.6	7.2	14.5
IODP-ICDP Exp. 364	40_2_0_3	617.67	IODP-ICDP Exp. 364 Bedded suevite unit	Suevite	70.47	Calcite vein	PIM2-4	3	-9.97	20.63	0.10	0.5461	0.0180	42.6	5.5	11.0
IODP-ICDP Exp. 364	44_1_46_48	628.90	IODP-ICDP Exp. 364 Graded suevite unit	Suevite	31.35	Bulk powder	PIM3-1	3	-11.52	19.03	-0.79	0.4431	0.0177	94.6	8.4	16.8
IODP-ICDP Exp. 364	48_1_22_24	640.86	IODP-ICDP Exp. 364 Graded suevite unit	Suevite	30.00	Bulk powder	PIM2-5	3	-10.92	19.65	0.34	0.4384	0.0174	97.6	8.4	16.9
IODP-ICDP Exp. 364	54_1_64_66	659.58	IODP-ICDP Exp. 364 Graded suevite unit	Suevite	26.07	Bulk powder	PIM3-3	3	-11.70	18.85	-0.86	0.3877	0.0299	135.9	20.9	41.9
IODP-ICDP Exp. 364	59_2_74_76	675.91	IODP-ICDP Exp. 364 Graded suevite unit	Suevite	35.07	Bulk powder	PIM2-7	3	-9.80	20.81	1.72	0.4742	0.0174	76.3	7.1	14.2
IODP-ICDP Exp. 364	67_1_13_15	690.59	IODP-ICDP Exp. 364 Graded suevite unit	Suevite	48.65	Bulk powder	PIM3-5	3	-6.56	24.15	1.82	0.5650	0.0178	35.3	5.1	10.1
IODP-ICDP Exp. 364	44_1_46_48	628.90	IODP-ICDP Exp. 364 Graded suevite unit	Suevite		Carbonate clast	PIM3-2	3	-9.46	21.16	0.03	0.4707	0.0177	78.2	7.4	14.7
IODP-ICDP Exp. 364	48_1_22_24	640.86	IODP-ICDP Exp. 364 Graded suevite unit	Suevite		Carbonate clast	PIM2-6	3	-13.50	16.99	0.08	0.4397	0.0176	96.8	8.5	17.0

IODP-ICDP Exp. 364	59_2_74_76	675.91	IODP-ICDP Exp. 364 Graded suevite unit	Suevite	97.61	Carbonate clast	PIM2-8	3	-8.17	22.49	1.93	0.5123	0.0176	57.2	6.1	12.2
IODP-ICDP Exp. 364	67_1_13_15	690.59	IODP-ICDP Exp. 364 Graded suevite unit	Suevite		Carbonate clast	PIM3-6	3	-5.96	24.77	2.33	0.5412	0.0181	44.6	5.6	11.3
IODP-ICDP Exp. 364	81_3_30_32	710.29	IODP-ICDP Exp. 364 Non-graded suevite unit	Suevite	36.93	Bulk powder	PIM2-9	3	-10.73	19.85	0.93	0.5063	0.0175	60.0	6.2	12.4
IODP-ICDP Exp. 364	83_1_115_119	713.23	IODP-ICDP Exp. 364 Non-graded suevite unit	Suevite	70.96	Bulk powder	PIM2-10	3	-10.13	20.47	2.24	0.3850	0.0173	138.3	11.4	22.7
IODP-ICDP Exp. 364	84_3_70_72.5	715.52	IODP-ICDP Exp. 364 Non-graded suevite unit	Suevite	46.53	Green matrix	PIM2-11	3	-13.57	16.92	1.09	0.3516	0.0175	171.7	14.5	28.9
IODP-ICDP Exp. 364	87_2_73_75	721.45	IODP-ICDP Exp. 364 Upper Impact melt rock	Impact melt rock	79.22	Bulk powder	PIM2-16	4	-10.16	20.44	1.14	0.3473	0.0151	176.6	12.4	24.8
IODP-ICDP Exp. 364	89_1_57_59	726.21	IODP-ICDP Exp. 364 Upper Impact melt rock	Impact melt rock	24.57	Bulk powder	PIM2-19	2	-16.34	14.06	-3.56	0.4887	0.0216	68.6	8.6	17.3
IODP-ICDP Exp. 364	84_3_117.5_119.5	716.00	IODP-ICDP Exp. 364 Upper Impact melt rock	Impact melt rock	94.82	Carbonate clast	PIM1-1	4	-6.39	24.32	0.42	0.5458	0.0149	42.8	4.3	8.6
IODP-ICDP Exp. 364	86_1_9.5_11.5	717.74	IODP-ICDP Exp. 364 Upper Impact melt rock	Impact melt rock	96.37	Carbonate clast	PIM2-13	3	-8.42	22.23	-0.19	0.4832	0.0175	71.5	6.9	13.7
IODP-ICDP Exp. 364	84_3_117.5_119.5	716.00	IODP-ICDP Exp. 364 Upper Impact melt rock	Impact melt rock	49.60	Green matrix	PIM2-12	3	-10.32	20.27	-0.51	0.5000	0.0178	63.0	6.5	13.0
IODP-ICDP Exp. 364	87_2_56_58	721.28	IODP-ICDP Exp. 364 Upper Impact melt rock	Impact melt rock	41.56	Green matrix	PIM2-15	2	-9.04	21.59	-0.09	0.5077	0.0216	59.3	8.0	16.0
IODP-ICDP Exp. 364	87_2_73_75	721.45	IODP-ICDP Exp. 364 Upper Impact melt rock	Impact melt rock	88.03	Green matrix	PIM1-2	3	-9.67	20.94	1.59	0.2627	0.0172	326.6	33.4	66.8
IODP-ICDP Exp. 364	87_2_87_91	721.59	IODP-ICDP Exp. 364 Upper Impact melt rock	Impact melt rock	56.77	Green matrix	PIM2-17	3	-11.81	18.73	-1.01	0.4674	0.0174	80.1	7.3	14.6

IODP-ICDP Exp. 364	89_1_57_59	726.21	IODP-ICDP Exp. 364 Upper Impact melt rock	Impact melt rock	68.28	Green matrix	PIM1-3	3	-15.43	15.00	-2.43	0.4999	0.0172	63.1	6.2	12.5
IODP-ICDP Exp. 364	92_3_12_16	737.51	IODP-ICDP Exp. 364 Upper Impact melt rock	Impact melt rock	47.70	Green matrix	PIM1-4	2	-19.00	11.32	-2.17	0.4045	0.0209	121.9	12.7	25.4
Certified reference material	ETH-2							77				0.2076	0.0055			

SI References

See reference list in main text.
